# Carbazole Framework as Functional Scaffold for the Design of Synthetic Receptors

**DOI:** 10.1002/chem.202500126

**Published:** 2025-04-03

**Authors:** Alessio Carioscia, Debora Iapadre, Elena Incerto, Jonathan Di Pietro, Luisa Giansanti, Fabio Pesciaioli, Armando Carlone

**Affiliations:** ^1^ Department of Physical and Chemical Sciences Università degli Studi dell'Aquila L'Aquila 67100 Italy; ^2^ Institut de Science et d'Ingénierie Supramoléculaires (ISIS) University of Strasbourg & CNRS Strasbourg 67000 France; ^3^ INSTM, Consorzio Nazionale per la Scienza e Tecnologia dei Materiali L'Aquila Italy

**Keywords:** carbazole, host–guest systems, noncovalent interactions, receptors, supramolecular chemistry

## Abstract

Carbazole serves as a prominent framework in the design of synthetic receptors, being a valuable scaffold for supramolecular chemistry, thanks to its planarity, fluorescence and versatility. This review provides a comprehensive analysis of notable examples of carbazole‐based receptors, highlighting the impact of structural modifications on binding affinity and selectivity toward different guests.

## Graphical abstract

Carbazole‐based receptors play a crucial role in supramolecular chemistry, due to their unique geometry, photophysical properties, strong hydrogen bonding features, and versatility. This review examines prominent examples, focusing on how structural modifications shape binding affinity and selectivity for various guests, showcasing the potential of carbazole scaffolds to underpin innovation in synthetic receptor design.

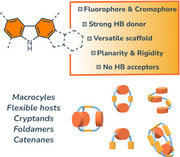

## 1 Introduction

**Figure 1:**
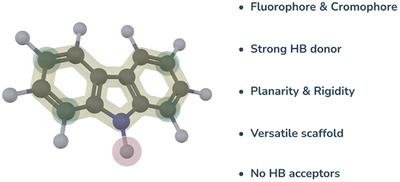
Carbazole scaffold and its properties.

The development of synthetic receptors for small molecules is an area of significant interest for supramolecular chemistry. The study of host–guest complexes has shown terrific applications in the fields of optical sensing of cations and anions,^[^ ^1^ ^]^ molecular detection,^[^ ^2^ ^]^ cellular targeting,^[^ ^3^ ^]^ drug delivery and in general for the disruption of communication mechanisms within the cells,^[^ ^4^ ^]^ as well as in catalysis.^[^ ^5^ ^]^ For this purpose, several building blocks and molecular scaffolds have been employed, such as ureas, thioureas,^[^ ^5^, ^6^, ^7^, ^8^ ^]^ squaramides,^[^ ^9^, ^10^ ^]^ triazoles,^[^ ^11^, ^12^, ^13^ ^]^ imidazoles,^[^ ^14^ ^]^ pyrroles,^[^ ^15^ ^]^ triphenylenes,^[^ ^16^ ^]^ steroids,^[^ ^17^, ^18^ ^]^ cyclodextrines, cucurbituriles and calixarenes.^[^ ^19^, ^20^, ^21^, ^22^, ^23^, ^24^ ^]^ Among these, carbazole received prominent attention as a suitable building block for the construction of synthetic receptors. Indeed, carbazole represents an ideal functional framework thanks to different features (Figure 1). Carbazole derivatives are generally chromophores and exhibit fluorescent properties, allowing the development of fluorescent sensors ^[^ ^25^, ^26^ ^]^ and colorimetric probes.^[^ ^27^, ^28^ ^]^ The optical properties of carbazole‐based receptors undergo significant changes upon guest binding; fluorescence quenching or enhancement can be observed depending on the specific guest. In this regard, a comprehensive review highlights the photophysical properties and biological application of carbazole‐based fluorescent probes in bioimaging.^[^ ^29^ ^]^ Furthermore, its rigid skeleton and planar geometry facilitate preorganization of the host molecule^[^ ^30^ ^]^ and allow for the formation of high order supramolecular aggregates through π‐stacking interactions. Carbazole possess a strong and integrated hydrogen bond donor (*α*_2_^H^ = 0.47 on the Abraham's scale), making it the most potent among NH‐based heterocycles commonly employed as H‐bond donors, such as imidazole (*α*_2_^H^ = 0.42), pyrrole (*α*_2_^H^ = 0.41), benzimidazole (*α*_2_^H^ = 0.42), and indole (*α*_2_^H^ = 0.44).^[^ ^31^, ^32^ ^]^ Interestingly, carbazole does not possess hydrogen bonding acceptor moieties, thus preventing self‐association which would hamper the binding affinity, as extensively reported for thiourea derivatives in asymmetric catalysis.^[^ ^33^ ^]^ It is crucial to emphasize that carbazole integrates a fluorophore, a chromophore and a strong hydrogen bonding donor, besides being an advantageous structural framework. Finally, it is a particularly versatile scaffold for derivatizations. Synthetic receptors obtained from the functionalization in positions 1 and 8, along with other derivatization on the aromatic rings, are particularly interesting for the synthesis of receptors featuring multiple moieties capable of establishing additional noncovalent interactions (e.g., hydrogen bonding donors, halogen bonding acceptors). Indeed, after prefunctionalization, this kind of derivatizations allows a facile access to amide, thioamide, urea, thiourea or sulphonamide carbazole‐derivatives which exhibit high and tunable binding properties.

Although several reviews highlight certain examples of carbazole‐based receptors,^[^ ^34^, ^35^, ^36^, ^37^ ^]^ a more comprehensive work that encompasses all recent literature on this class of receptors might contribute to the design of more powerful hosts both in terms of affinity and selectivity. This Review aims to describe the most recent and relevant examples of carbazole‐based receptors, highlighting structural features to providing insights into the relationships between the structure and the binding affinity/selectivity. This work will be divided into two parts. The first section will cover the most important literature regarding the use of carbazole‐based receptors in binding of anions, with a special emphasis on their application. The second part will cover the applications of carbazole‐based synthetic receptors in binding of neutral organic compounds of biological interest (e.g., carbohydrates, xanthines and others).

## 2 Carbazole‐Based Receptors for Anions

Anions possess a prominent role in several biological processes. In particular, the chloride anion has been extensively studied as guest for synthetic receptors for its biological relevance in the regulation of transmembrane flux.^[^ ^38^ ^]^ The disruption of ion channels is related to several diseases, namely channelopathies,^[^ ^39^, ^40^ ^]^ such as cystic fibrosis,^[^ ^41^, ^42^ ^]^ epilepsy,^[^ ^43^ ^]^ and dysfunction of the vestibular system.^[^ ^44^ ^]^ The development of synthetic carriers for biologically relevant anions is therefore interesting for therapeutical applications.^[^ ^45^, ^46^ ^]^ Furthermore, other anions, such as nitrate, sulfate and phosphate, have been recognized as pollutants both in soil and water.^[^ ^47^, ^48^ ^]^ Therefore, the development of host–guest systems for the binding of these anions is crucial.^[^ ^7^ ^]^

### 2.1 1,8‐Diaminocarbazole Platform

**Figure 2:**
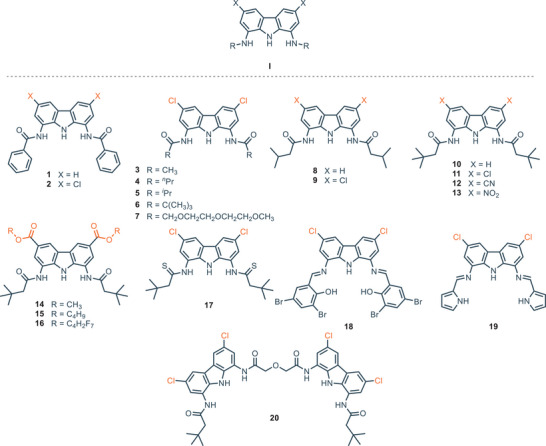
1,8‐diamino‐3,6‐dichlorocarbazole scaffold, mono‐ and bis‐amido, and imino‐carbazoles receptors.

In 2004, Jurczak and coworkers. reported the first synthetic receptor for anions based on a 1,8‐diamino‐3,6‐dichlorocarbazole scaffold **I** (Figure 2). The significance of this work lies in the demonstration of the versatility of this scaffold, paving the way for the development of a new family of anion receptors. The authors show the higher affinity of the receptors **2** and **4** for polyatomic anions (e.g., dihydrogen phosphate and acetate) compared with chloride, since the cleft appears to be too wide to locate the small and monoatomic Cl^−^.^[^ ^25^ ^]^ The synthetic protocol involves the dichlorination of carbazole at positions 3 and 6, followed by nitration at positions 1 and 8 using absolute nitric acid. A catalytic hydrogenation step then enables the preparation of 1,8‐diamino‐3,6‐dichlorocarbazole. There are some challenges related to the synthesis of these platforms. Generally, harsh conditions are required for the nitration of the 1,8‐diaminocarbazole precursor and limitations are still present for the substitution of positions 3 and 6 of the carbazole skeleton. Substitutions in these positions are used to modulate carbazole properties like binding affinity, solubility or fluorescence response. *t*‐Butyl groups are commonly used, which may additionally improve the solubility of desired receptor. Nevertheless, synthetic problems occur since *t*‐butyl groups are introduced in the first step and can undergo partial cleavage during the following steps. After the short synthesis of the dehalogenated 1,8‐diaminocarbazole developed by Chmielewski,^[^ ^49^ ^]^ several groups reported the synthesis of mono‐ and bis‐amido and imino carbazoles based receptors (Figure 2).^[^ ^26^, ^27^, ^28^, ^50^, ^51^, ^52^, ^53^, ^54^, ^55^, ^56^, ^57^ ^]^ Some of these studies have already been extensively covered by other review‐type articles.^[^ ^34^, ^35^ ^]^ For this reason, this review will mainly focus on the most recent advances in this topic. Most of the receptors shown in Figure 2, whose association constants with various anions are shown in Table 1, demonstrate a pronounced affinity for oxoanions in DMSO‐d_6_ + 0.5% H_2_O (in particular for carboxylates and dihydrogen phosphate). The reduced binding affinity of halide anions (e.g., Cl^−^) compared to oxoanions can be attributed to the lower basicity of chloride and its neither hard nor soft character compared to the oxoanions under study. Additionally, a structural mismatch between the receptor and the chloride anion hinders effective interactions with the receptor's proton sites.^[^ ^25^ ^]^

**Table 1:** Association constants (m ^−1^) for compounds **1**–**20** toward anionic guests. Unless specified, the titrations are performed via UV–visible in DMSO + 0.5% H_2_O.

Receptor	K_A_(acetate)	K_A_(benzoate)	K_A_(H_2_PO_4_^−^)	K_A_(F^−^)	K_A_(Cl^−^)	K_A_(Br^−^)	K_A_(SO_4_^−2^)	Ref.
**1**	‐	5.73 × 10^2^	1.66 × 10^3^	‐	<10^[b]^	‐	‐	^[^ ^51^ ^]^
**2**	‐	1.79 × 10^3^	6.98 × 10^3^	‐	14^[b]^	‐	‐	^[^ ^51^ ^]^
**3**	‐	7.55 × 10^3^	1.21 × 10^5^	‐	75^[b]^	‐	‐	^[^ ^51^ ^]^
**4**	‐	1.60 × 10^4^	9.62 × 10^4^	‐	109^[b]^	‐	‐	^[^ ^51^ ^]^
**5**	‐	1.31 × 10^4^	1.21 × 10^5^	‐	131^[b]^	‐	‐	^[^ ^51^ ^]^
**6**	‐	2.01 × 10^3^	4.71 × 10^3^	‐	56^[b]^	‐	‐	^[^ ^51^ ^]^
**7** ^[a]^	‐	‐	‐	‐	‐	‐	10^4^	^[^ ^56^ ^]^
**8**	‐	4.43 × 10^3^	1.10 × 10^4^	‐	42^[b]^	‐	‐	^[^ ^51^ ^]^
**9**	‐	2.18 × 10^4^	9.68 × 10^4^	‐	123^[b]^	‐	‐	^[^ ^51^ ^]^
**10**	‐	4.65 × 10^3^	1.02 × 10^4^	‐	48^[b]^	‐	‐	^[^ ^51^ ^]^
**11**	‐	2.90 × 10^4^	8.32 × 10^4^	‐	159^[b]^	‐	‐	^[^ ^51^ ^]^
**12** ^[b]^	‐	deprotonation	2.51 × 10^5^	‐	309	‐	‐	^[^ ^27^ ^]^
**13** ^[b]^	‐	deprotonation	deprotonation	‐	347	‐	‐	^[^ ^27^ ^]^
**14**	deprotonation	2.14 × 10^4^	8.71 × 10^4^	‐	123^[b]^	‐	deprotonation	^[^ ^26^ ^]^
**15**	‐	1.58 × 10^4^	5.25 × 10^4^	‐	‐	‐	‐	^[^ ^26^ ^]^
**16**	‐	1.02 × 10^4^	1.32 × 10^5^	‐	‐	‐	‐	^[^ ^26^ ^]^
**17** ^[b]^	‐	‐	‐	‐	67	‐	‐	^[^ ^60^ ^]^
**18** ^[c]^	1.79 × 10^4^	‐	2.13 × 10^4^	1.08 × 10^4^	n.d	‐	‐	^[^ ^28^ ^]^
**19** ^[d]^	2.24 × 10^4^	‐	6.17 × 10^4^	‐	2.57 × 10^3^	3.16 × 10^3^	‐	^[^ ^52^ ^]^
**20**	‐	6.02 × 10^4^	2.19 × 10^5^	‐	758^[d]^	‐	^[e]^	^[^ ^57^ ^]^

^[a]^ Measured via ^1^H NMR titrations in DMSO‐d_6 _+ 10% H_2_O.

^[b]^ Measured via ^1^H NMR titrations in DMSO‐d_6 _+ 0.5% H_2_O.

^[c]^ Measured in DMSO.

^[d]^ Measured in acetonitrile.

^[e]^ Too high to be measured. n.d. = The association constant could not be determined.

**Figure 3:**
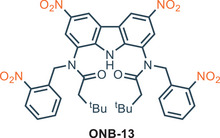
Procarrier **ONB‐13** which requires both light and acidic pH to become active.

The functionalization of positions 3 and 6 on the carbazole ring with electron‐withdrawing groups generally increases the binding affinities because the NH can act as stronger hydrogen bond donor. Notably, a significant difference between the 1:1 association constants is observed comparing receptor **1** (*K*_A_ = 5.73 × 10^2^ m ^−1^ for benzoate; *K*_A_ = 1.66 × 10^3^ m ^−1^ for dihydrogen phosphate) ^[^ ^51^ ^]^ with its chlorinated analogue **2** (*K*_A _= 1.79 × 10^3^ m ^−1^ for benzoate; *K*_A _= 6.98 × 10^3^ m ^−1^ for dihydrogen phosphate).^[^ ^51^ ^]^ A similar trend is evident when receptor **8** (*K*_A _= 4.43 × 10^3^ m ^−1^ for benzoate; *K*_A _= 1.10 × 10^4^ m ^−1^ for dihydrogen phosphate) is compared to its chlorinated counterpart **9** (*K*_A _= 2.18 × 10^4^ M^−1^ for benzoate; *K*_A _= 9.68 × 10^4^ m ^−1^ for dihydrogen phosphate) and when receptor **10** (*K*_A _= 4.65 × 10^3^ m ^−1^ for benzoate; K_A _= 1.02 × 10^4^ m ^−1^ for dihydrogen phosphate) is compared with receptor **11** (*K*_A _= 2.90 × 10^4^ m ^−1^ for benzoate; *K*_A _= 8.32 × 10^4^ m ^−1^ for dihydrogen phosphate).^[^ ^51^ ^]^ The highest affinity for dihydrogen phosphate with respect to benzoate could be ascribed to an higher structural complementarity with the host. It was observed that the non‐substituted receptor **10** exhibited an excellent fluorescent response compared to **11** even though the latter is able to discriminate different anions.^[^ ^51^ ^]^ Chmielewski and coworkers, in 2021, investigated receptors with strongly electron‐withdrawing groups at the 3,6 positions of the carbazole ring (**12** and **13**).^[^ ^27^ ^]^. The binding strength is linearly dependent on the electron withdrawing character of the substituents and is influenced by proton transfer from carbazole to the basic anion, suggesting an increase in the acidity of carbazole NH resulting from the introduction of strongly electron‐withdrawing groups.^[^ ^27^ ^]^ In 2024 the same group explored functionalization at the 3,6 positions with ester groups containing various alkoxy substituents (**14**, **15**, and **16**).^[^ ^26^ ^]^ Their findings revealed a high binding affinity with dihydrogen phosphate and benzoate. In addition, they demonstrated the deprotonation of receptor **12** in the presence of acetate and sulfate, while no deprotonation was observed with benzoate, in contrast with the behavior exhibited by receptors **12** and **13**.^[^ ^26^ ^]^ This phenomenon can be attributed to the higher electron‐withdrawing nature of ─CN and ─NO_2_ groups in receptors **12** and **13** compared to the esters groups present in receptors **14**, **15**, and **16**. Overall, the alkoxy groups of these ester functionalized receptors have a minimal impact on binding affinity.^[^ ^26^ ^]^ Later, compound **13** was employed as a chloride transporter across the lipid bilayer, functioning as a Cl⁻/NO₃⁻ exchanger. Interestingly, its activity was found to be strongly influenced by pH, with the highest transport rate observed at acidic pH, while significantly decreasing at neutral pH.^[^ ^58^ ^]^ This behavior is due to the electrostatic repulsion between the carbazole anion and the negative charged guest. The pH dependence was then exploited for the development of a dual‐stimuli responsive anion transporter (**ONB‐13**) based on the structure of **13** and appended with the light‐cleavable *o*‐nitrobenzyl group (ONB). (Figure 3) This carrier is inactive in its native form, and it requires two orthogonal stimuli—light and acidic pH—to achieve maximum activity.

Structural variations of amide R groups, whether aromatic or aliphatic, also impact anion binding affinities. Interestingly, receptors with aliphatic groups show stronger binding compared to their aromatic counterparts, despite the additional hydrogen bonds between ortho CH protons of the phenyl ring (of the receptors **1** and **2**) and the anion. This discrepancy may result from the higher energetic cost associated with disrupting intramolecular hydrogen bonds in aromatic substituents upon the anion binding.^[^ ^51^ ^]^ Comparing the activity of receptors with aliphatic substituents (**3**, **4**, **5**, **6**, **9**, and **11**), it becomes evident that the binding affinity for anions is predominantly affected by subtle structural variations within the aliphatic chain.^[^ ^51^ ^]^ For instance, the introduction of methyl groups at the β carbon (e.g., receptor **9**, *K*_A _= 2.18 × 10^4^ m ^−1^ for benzoate)^[^ ^51^ ^]^ enhances binding affinity compared to receptor **4** (e.g., *K*_A _= 1.60 × 10^4^ m ^−1^ for benzoate), while the addition of a methyl group at the α carbon (e.g., receptor **6**, *K*_A _= 2.01 × 10^3^ m ^−1^ for benzoate) decreases the binding affinity compared to receptor **5** (e.g., *K*_A _= 1.31 × 10^4^ m ^−1^ for benzoate).^[^ ^51^ ^]^ Nevertheless, the addition of a methyl group at the α position enhances the solubility of the receptor in organic solvents. This is likely attributed to the increased steric bulk, which not only hinders anion binding but also interferes with receptor–receptor interactions.^[^ ^51^ ^]^ The fact that enhanced steric bulk introduced with *t*‐butyl groups at positions 3 and 6 on the carbazole ring increases hydrophobicity and steric hindrance, which in turn improves solubility in organic solvents, may explain the popularity of scaffolds derivatized in these positions. On the other hand, receptors with short linear alkyl chains at positions 3 and 6, such as *n*‐butyl groups, are rarely used, likely because they do not offer advantages in terms of solubility or binding. *t*‐butyl groups have the same effect at position 2 and 5. In particular, *n*‐hexyl groups confer a higher solubility compared to the *t*‐butyl ones.^[^ ^59^ ^]^

Thioamide receptors, such as receptor **17**, demonstrate lower binding affinities compared to their amide analogues. This decreased affinity can likely be attributed to the larger size of the sulfur atom relative to oxygen, which induces twisted geometry, thus influencing the interaction with anions.^[^ ^60^ ^]^ Nevertheless, the thioamide groups show higher solubility than the amide analogues, which suffer from poor solubility. For these reasons, **17** is a very efficient anionophore for the transport of chloride, oxoanions (e.g., carboxylates and organic phosphates) and non‐steroidal anti‐inflammatory drugs (NSAIDs) like aspirin across lipid membranes.^[^ ^61^ ^]^ Diamide and dithioamide–carbazole receptors **11**–**13** and **17** also act as active transporters of bicarbonate anion across lipid membrane.^[^ ^62^ ^]^ In 2022, exploiting a direct detection method based on an europium complex assay,^[^ ^63^ ^]^ the effective transport mechanism of bicarbonate across a lipid membrane was established, determining that two different processes of transport occur in the presence of **11**–**13** and **17**: one is the indirect transport caused by the spontaneous diffusion of CO_2_, coupled with pH equilibration via H^+^Cl^−^ symport by receptors **11**–**13** and **17**; the other is the direct bicarbonate anion transport by these receptors via HCO_3_^−^/Cl^−^ antiport. These two processes, as expected, are remarkably dependent on receptor concentration, but they are assumed to be independent of each other. **11** does not show active transport HCO_3_^−^ anions; on the other hand, the presence of electron‐withdrawing groups on **12** and **13** enhances the transporter activity. The best performance is displayed by dithioamide **17** which confirms to be a potent anionophore for oxoanions. Diamide **11–13** and dithioamide **17** also showed antimicrobial activity in accordance with their efficiency as anion transporter.^[^ ^62^ ^]^ The dithioamide receptor **17** was also effectively employed in the transport of amino acids (AAs) across lipid bilayers (see Section 3). Imine carbazole‐based receptor **18** additionally employs phenolic protons for binding interactions and exhibits a high binding affinity in DMSO for dihydrogen phosphate (*K*_A _= 2.13 × 10^4^ m ^−1^) and acetate (*K*_A _= 1.79 × 10^4^ m ^−1^).^[^ ^28^ ^]^ Notably, fluoride is bound effectively (*K*_A _= 1.08 × 10^4^ m ^−1^), unlike other halide anions. The greater affinity for dihydrogen phosphate compared to acetate can be attributed to the tetrahedral conformation, which facilitates a more favorable interaction with the receptor. This is likely due to a better structural complementarity between the anion and the receptor, resulting in an increased number of interactions.^[^ ^28^ ^]^

The imine carbazole‐based receptor **19** also comprises pyrrolic protons that can assist in the binding process. This receptor shows a strong binding affinity in acetonitrile for various anions, including dihydrogen phosphate (*K*_A _= 6.17 × 10^4^ m ^−1^), acetate (*K*_A_ = 2.24 × 10^4^ m ^−1^), chloride (*K*_A _= 2.57 × 10^3^ m ^−1^), and bromide (*K*_A _= 3.16 × 10^3^ m ^−1^).^[^ ^52^ ^]^ The interaction is stronger with the first two anions (dihydrogen phosphate and acetate), consistent with their higher basicity. However, despite acetate being more basic, dihydrogen phosphate is bound more efficiently. This can be attributed to the ability of dihydrogen phosphate groups to interact with the lone pairs of the imine nitrogens. Specifically, the acidic protons of this guest act as hydrogen bond donors to the imine nitrogens of the receptor.^[^ ^52^ ^]^

**Figure 4:**
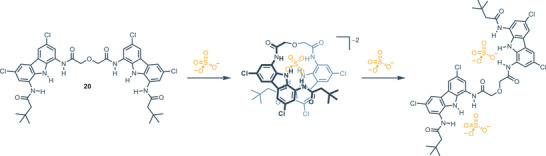
Structural features of the complex **20** with sulfate ion.

**Figure 5:**
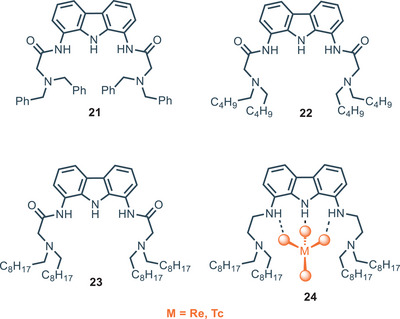
Amido and amino carbazole‐based receptors for the extraction of ReO_4_^−^/TcO_4_^−^.

**Figure 6:**
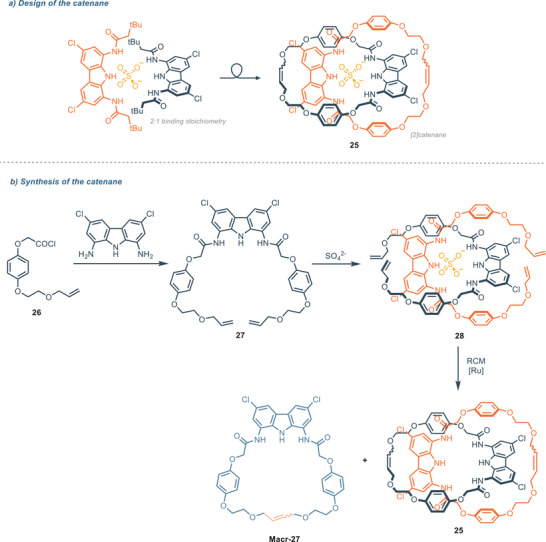
a) Design of [2]catenane exhibiting strong affinity and selectivity for sulfate in DMSO‐d_6_ + 10% H_2_O; b) Synthesis of the [2]catenane via sulfate tempated RCM.

Receptors **11** and **7** form robust and orthogonal 2:1 complexes with SO_4_^2−^ in DMSO‐d_6_ + 10% H_2_O, even in the presence of a large amount of water (10%).^[^ ^56^ ^]^ This orthogonal assembly pattern was later utilized for the construction of catenanes as shown in Figure 6a.^[^ ^64^ ^]^ In case of receptor **2**, in the 2:1 complex with sulfate, the formation of orthogonal complexes does not occur. Instead, the two receptor molecules adopt a biplanar arrangement, driven by the establishment of π─π stacking interactions due to the presence of aromatic groups.^[^ ^56^ ^]^ The dicarbazole receptor **20** exhibits selectivity for the sulfate anion, demonstrating high association constants in both 1:1 and 1:2 complex stoichiometries.^[^ ^57^ ^]^ Indeed, in both instances, the association constants could not be determined due to the exceptionally high affinity. Specifically, upon the addition of one mole of sulfate, receptor **20** binds the anion with both carbazole moieties. However, in presence of excess sulfate, the two carbazole units bind two different sulfateanions, rather than sharing the same one. Figure 4 depicts the structural features of the complex under examination. ^[^ ^57^ ^]^ Designing an alternative method for the treatment of TcO_4_^−^ in nuclear water waste, amidic and aminic carbazole scaffolds have also been inserted in the synthesis of receptors able to extract ReO_4_^−^ and TcO_4_^−^ from acidic aqueous solution.^[^ ^55^ ^]^ The method developed consists of a liquid–liquid extraction in which the organic phase is filled with diamide and diamino carbazole‐based receptors equipped with tertiary amines as appendages (Figure 5). These receptors can extract ReO_4_^−^/TcO_4_^−^ from acidic solutions of HNO_3_ at various pH. Among all the receptors tested, **24** seems to display the best activity in comparison with the amide‐based ones **21**–**23**. Amide‐based receptors **21**–**23** show an intramolecular hydrogen bond, formed between carbazole NH and C═O groups, that brings to anti‐preorganization because of the occlusion of the cavity intended for the anion. Moreover, due to intramolecular H‐bonding, the two amide NH sites point out on different planes, hence their resulting interactions with the anion are less efficient.

As expected, the extraction capacity of **24** is higher with respect to **21**–**23** due to the fact that in acidic condition the protonation of the NH brings about a positive electrostatic attraction with the negatively charged guest. It is further influenced by the presence of competitive anions, e.g., NO_3_^−^. Increasing the concentration of NO_3_^−^, the affinity of **23** for ReO4^−^ gradually decreased. Nevertheless, the capacity of extraction of **24** is higher toward TcO_4_^−^ than toward ReO_4_^−^, as a consequence of the lower hydration energy of TcO_4_^−^.

**Figure 7:**
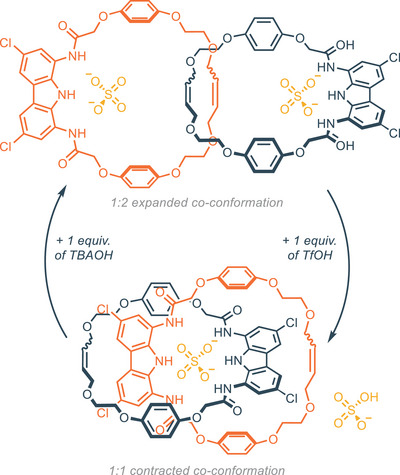
The co‐conformation cycle for the [2]catenane triggered by acidic and basic conditions. Adapted from ref. [64].

In 2023, Chmielewski and coworkers developed a charge‐neutral [2]catenane **25** (Figure 6a) based on a 1,8‐diamidocarbazole architecture via a sulfate‐templated ring closing metathesis (RCM) (Figure 6b),^[^ ^64^ ^]^ starting from the observation that simple diamidocarbazole **11** exhibits strong affinity toward sulfate in a 2:1 (receptor:anion) stoichiometry, where the two ligands are arranged in an orthogonal fashion.^[^ ^65^ ^]^ The [2]catenane **25** showed excellent affinity for SO_4_^2−^ in DMSO‐d_6_/H_2_O 9:1 (*K*_A _= 7.76 × 10^5^ m ^−1^ via NMR). Furthermore, the 1:1 association constant of [2]catenane **25** toward dihydrogen phosphate and benzoate are 95 and 3400 times lower respectively, indicating outstanding selectivity. Compound **25** binds sulfate with one order of magnitude higher than simple diamidocarbazole **11**, and 2.5 times than bis(carbazole) receptor **20**. Notably, the macrocyclic compound **Macr‐27** (obtained as a side product in the RCM reaction) and the acyclic precursor **27** have an association constant respectively 20 and 10 times lower than the catenane. The 1:1 complex (namely contracted co‐conformation) and the 1:2 complex (namely expanded co‐conformation) can be easily switched through acid‐base triggering, as previously demonstrated for compound **11** (Figure 7).^[^ ^66^ ^]^ Upon the addition of 1 equiv. of triflic acid to a 2 mm solution of [2]catenane and TBA_2_SO_4_ (2 equiv.) in DMSO‐d_6_, protonation of 1 equiv. of sulfate occurs, shifting the conformation from the expanded to the contracted one. The addition of 1 equiv. of tetrabutylammonium hydroxide (TBAOH) allows for the opposite conformation change. This co‐conformation cycle can be repeated up to 8 times.

**Figure 8:**
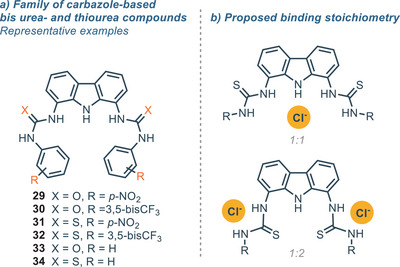
a) Family of urea and thiourea derivatives; b) Different binding stoichiometries.

1,8‐diaminocarbazoles have also been effectively conjugated with urea‐ and thiourea‐ motifs, which are known to be privileged hydrogen bonding donor moieties for their peculiar properties.^[^ ^5^, ^6^, ^7^, ^8^ ^]^ In fact, the introduction of additional acidic NH protons that could synergistically interact with the guest is thought to increase the binding affinity.^[^ ^53^ ^]^ Additionally, the urea linker allows for the introduction of different diaminocarbazole moieties, exploiting the formation of macrocyclic compounds, thus defining shape‐persistent cavities that could further assist in the binding process.^[^ ^67^ ^]^ In 2021, Gale and coworkers reported a series of carbazole‐based bis ureas‐ and thioureas, with the most relevant, compounds **29**–**34**, shown in Figure 8a.^[^ ^68^ ^]^ This family of compounds displayed moderate affinity toward Cl^−^, with the highest values reported for compounds bearing an aromatic moiety with EWG groups. Moreover, the urea‐derivative compounds **29**–**30** showed a much higher association constant than their thiourea counterparts **31**–**32** (*K*_A_ = 100–300 m ^−1^ *versus* *K*_A_ = 60–120 m ^−1^ in DMSO‐d_6_ + 0.5% H_2_O). This difference is probably due to the distortion of planarity induced by the steric hindrance of the sulfur atom. Interestingly, the titration for the thiourea derivatives revealed that a 1:2 (receptor:anion) stoichiometry is observed for high concentration of Cl^−^ (Figure 8b). The moderate affinity toward Cl^−^ has been rationalized considering the large cavity of the 1,8‐diaminocarbazole scaffold, in accordance with other reports.^[^ ^25^ ^]^ It becomes clear that bigger, polyatomic anions are the favorite anionic guests for receptors based on the 1,8‐diaminocarbazole scaffold. Nevertheless, both the urea and the thiourea derivatives **33** and **34** proved to bind Cl^−^ better than their amide counterpart **1** (*K*_A_ = 122 m ^−1^ for **33** versus *K*_A_ = 67 m ^−1^ for **34 **versus *K*_A_ < 1 m ^−1^ for **1**). This is probably due to the higher number of hydrogen bonding donor when the (thio)urea moieties is present, even though the spatial orientation of these group is also important.

**Figure 9:**
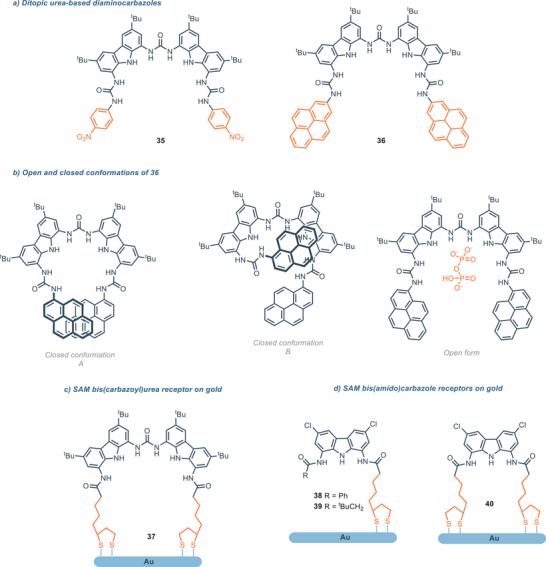
a) Ditopic urea‐based diaminocarbazoles; b) different conformations of receptor **36;** c) SAM bis(carbazoyl)urea‐based receptors receptor for HP_2_O_7_^3^ developed by Molina and coworkers; d) SAMs receptors for SO_4_^2−^ developed by Chmielewski and coworkers.

The effort directed toward the development of receptors that features a ditopic design is highlighted by the work of Molina and coworkers, where the ditopic 1,8‐diaminocarbazole‐based synthetic receptors **34** and **35** were synthesized and characterized (Figure 9a).^[^ ^69^ ^]^

These receptors feature two diaminocarbazole scaffolds appended with fluorogenic pyrenyl and chromogenic *p*‐nitrophenyl aromatic moieties; urea linkers are used to construct the molecular architectures. The eight well‐oriented NH groups define a cavity which is optimal for the detection of hydrogenopyrophosphate with high affinity and selectivity both in CD_3_CN and in competitive mixtures CD_3_CN/H_2_O. Here, a ditopic design seems to be optimal for the recognition of the large binuclear pyrophosphate, highlighting again the close relationship between the cavity size of the receptor and its corresponding optimal guest. Interestingly, the pyrenyl derivative **36** exhibits a large excimer emission band (*λ* = 496 nm) and two sharp bands (*λ* = 394 and 416 nm), consistent with the presence of a pyrene. The ratio between the excimer intensity and the monomer intensity (i.e., *I*_E_/*I*_M_) stays constant at different concentrations (10^−7^–10^−5^ m), indicating that the excimer formation happens in an intramolecular fashion. This is probably due to a π‐stacking interaction between the two pyrenyl groups (closed conformation A) or between a pyrenyl group and a carbazole moiety (closed conformation B). Interestingly, upon the addition of hydrogenopyrophosphate, the excimer band disappears, while the monomer band tends to increase. This behavior suggests that the inclusion of the anion inside the cavity inhibits intramolecular interaction, with preference for the corresponding open form which accommodates the guest. (Figure 9b).

**Figure 10:**
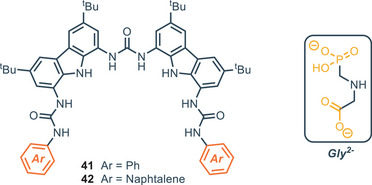
Ditopic receptors for binding of glyphosate.

From **35** or **36** it is possible the generation of self‐assembled monolayers (SAM) bis(carbazolyl)urea **37** on gold surface.^[^ ^65^ ^]^ **37** is a highly selective receptor and robust sensing probe for the surface plasmon resonance (SPR).^[^ ^70^ ^]^ Indeed, **37** shows high affinity for hydrogen pyrophosphate anion, HP_2_O_7_^3‐^ in buffered aqueous solution under physiological conditions (Figure 9c). SPR consists of a thin metal surface appropriately modified with a SAM bearing a molecular probe selective toward a particular species, having the advantage of operating in aqueous media.^[^ ^71^ ^]^ The high selectivity toward HP_2_O_7_^3^ makes receptor **37** an optimal sensor in the described systems. Interestingly, two different situations occur during the anion recognition in the operative conditions. At low anion concentration (HP_2_O_7_^3−^ in the order of 10^−10^–10^−7^ m), the most accessible receptors molecules only interact with the anions at the interface of the SAM. In this case, the association constant for HP_2_O_7_^3−^ is 4.95 × 10^3^ m ^−1^. On the other hand, at higher concentration (HP_2_O_7_^3−^ in the order of 10^−6^–10^−4^ m) the association constant is higher (*K*_A_ = 4.39 × 10^5^ m ^−1^) because in this case also the most buried receptors molecules at the interface can complex the anion. Furthermore, **37** proves to be selective toward HP_2_O_7_^3−^ in the presence of different anions, even phosphate and trivalent anions which display similar features with HP_2_O_7_^3−^. More recent SAMs of bis(amido)carbazole were developed by Chmielewski and coworkers for sulfate detection via vibrational spectroscopy techniques like surface infrared and Raman spectroscopy.^[^ ^72^ ^]^ SAM‐**38**–**40** are obtained from their analogues receptor **2** and **11** which showed high affinity and selectivity toward SO_4_^2−^. Sulfate affinity in acetonitrile depends on the substituents on the amide moieties. The affinity increases in the order: **38** < **40** < **39**. Nevertheless, no receptor‐anion interaction is detected in water for the latter SAMs receptors probably because the desolvation free energy required for the sulfate to be dehydrated and its competition with water for the binding site (absent in acetonitrile). Following the same general structure, in 2017 Leito and coworkers studied several hosts based on different hydrogen bonding scaffolds as anion receptors for binding the glyphosate dianion Gly^2−^ (Figure 10).^[^ ^73^ ^]^

Among all the different receptors studied, the bis(carbazolyl)urea derivatives **41** and **42** proved to be the most effective. In fact, in DMSO‐d_6_ + 0.5% H_2_O, compound **41** exhibits a *K*_A_ of 6.3 × 10^4^ m ^−1^, while compound **42** has a constant too high to be measured. With the aim to test the receptors in more competitive assays, the titration was repeated with 5% and 10% of water content. Compound **42** outperformed compound **41** both with 5% water (*K*_A_ = 3.16 × 10^6^ m ^−1^ for **42** versus *K*_A_ = 10^3^ m ^−1^ for **41**) and with 10% water (*K*_A_ = 3.16 × 10^5^ m ^−1^ for **42** versus *K*_A_ = 10^3^ m ^−1^ for **41**). Interestingly, **42** decreases its binding strength of 1 order of magnitude when going from 5% to 10% water, while compound **41** displays no change in its binding affinity in the same conditions. The optimized geometry for compound **42** indicates that the presence of the two naphthyl rings promotes a spiral‐like orientation of the NH groups around the phosponate moiety of the glyphosate triggering the tautomerization of the acidic proton on the PO_3_H^−^ to the NH group; in this conformation, the binding process between the receptor and Gly^2−^ occurs exclusively at the phosphonate. The binding data for compounds **29–36** and **41–42** are summarized in Table 2.

**Table 2:** Association constants (m ^−1^) for compounds **29**–**36** and **41–42** toward anionic guests. Unless specified, the titrations are performed via NMR in DMSO‐d_6_ + 0.5% H_2_O.

Receptor	K_A_(Cl^−^)	K_A_ (HP_2_O_7_^3−^)	K_A_(Gly^2−^)	Ref.
**29**	100	‐	‐	^[^ ^68^ ^]^
**30**	300	‐	‐	^[^ ^68^ ^]^
**31**	60	‐	‐	^[^ ^68^ ^]^
**32**	120	‐	‐	^[^ ^68^ ^]^
**33**	122	‐	‐	^[^ ^68^ ^]^
**34**	67	‐	‐	^[^ ^68^ ^]^
**35**	‐	3.16 × 10^6^^[^ ^[a]^, ^[b]^ ^]^	‐	^[^ ^69^ ^]^
**36**	‐	10^7^^[^ ^[a]^, ^[c]^ ^]^	‐	^[^ ^69^ ^]^
**41**	‐	‐	6.31 × 10^4^^[^ ^c^ ^]^	^[^ ^73^ ^]^
**42**	‐	‐	^[^ ^[c]^, ^[d]^ ^]^	^[^ ^73^ ^]^

^[a]^ Measured in CH_3_CN.

^[b]^ Measured via UV/vis absorption titrations.

^[c]^ Measured via fluorescence titration.

^[d]^ Association constant too high to be measured.

**Figure 11:**
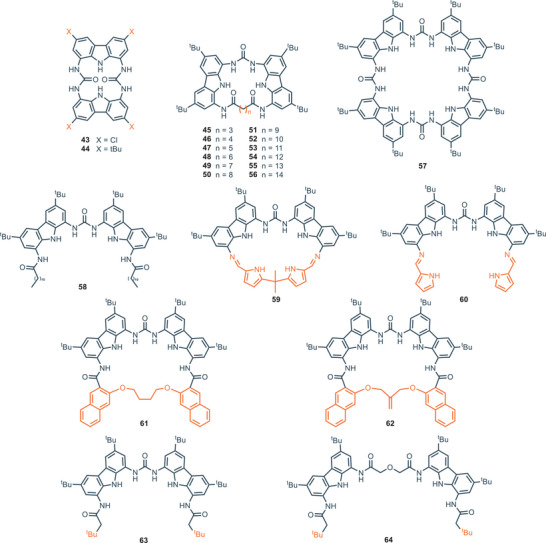
Cyclic and acyclic bis‐carbazolyl urea derivatives for binding of carboxylates.

The bis‐carbazolyl urea scaffold has also been employed in the design of macrocycles with different ring sizes (Figure 11). Kim and coworkers reported the cyclo‐bis‐(urea‐3,6‐dichlorocarbazole) **43** which exhibit good affinity for oxoanions, like acetate and dihydrogen phosphate (*K*_1:1_ = 1.5 × 10^3^ m ^−1^ and *K*_1:2_ = 1.5 × 10^5^ m ^−1^ for acetate; *K*_1:1_ = 1.4 × 10^3^ m ^−1^ and *K*_1:2_ = 1.8 × 10^5^ m ^−1^ for dihydrogen phosphate).^[^ ^74^ ^]^ Interestingly, X‐ray and computational analysis revealed that the binding occurs outside the cavity in a 1:2 fashion, with each anion interacting with the NH groups of one urea moiety. This explains the higher K_1:2_ with respect to K_1:1_ for the anions tested. Aiming to study the influence of the dimension of the cavity in the binding affinity, Leito and coworkers developed a series of carbazole‐based macrocycles **45**–**56** with different ring sizes, together with the derivative **57**, which exhibits a macrocyclic structure featuring 12 hydrogen bonding donor moieties, and the corresponding open‐chain analogue **58**.^[^ ^75^ ^]^ Computational modeling of these receptors versus various carboxylate anions showed that for the small macrocycles **45**–**47** preferential intramolecular hydrogen bonding is present. In fact, those macrocycles are too small to accommodate an anion inside their cavity. This was confirmed by the evaluation of their binding properties. A general trend was observed, where the association constant toward several tetrabutylammonium (TBA) salts of carboxylic acids was observed to be proportional with the ring size of the macrocycle, until it reaches a plateau for the receptors **49**–**51** (─CH_2_─: 7–9). A further increase in the methylene linker length has a negative effect on the binding affinity, which slowly decreases because the higher flexibility reduces the preorganization of the host. The open‐chain analogue **58** also competes well with other cyclic structures. In this case, macrocyclic structures did not prove to be significantly superior to more flexible open‐chain structures. Surprisingly, the rigid macrocycle **57**, which features 12 hydrogen bonding donors, performed much worse than other methylene‐bridged macrocycles (**44**–**56**). Although the authors do not provide an explanation for this phenomenon, it is important to consider that the general paradigm “higher rigidity leads to better binding” is not always true. Instead, binding strength strongly depends on the geometry of the cavity and the specific guest analyzed. In this case, the computed lowest‐energy conformations clearly indicate that the cavity is too large to accommodate small carboxylates. As a result, depending on the guest, the interaction involves only 4 to 7 of the 12 potentially available hydrogen‐bonds available. It is therefore crucial to carefully tailor the receptor based on the desired guest, in order to maximize the structural match. The same group, in 2022, reported the synthesis and binding characterization of compounds **59**–**64** as carboxylate‐binding receptors.^[^ ^76^ ^]^ These molecules feature unusual connection motifs, such as imines and ether bridges. All the receptors were tested toward a set of carboxylate anions (acetate, benzoate, sorbate, formate, and lactate) and the general trend is in line with the observation that the higher basicity of the anion correlates with higher binding strength.^[^ ^77^ ^]^ The cyclic imine‐derivative **59** proved to be the most powerful toward recognition for most of the anions. In particular, compound **59** strongly binds acetate in DMSO‐d_6_ + 0.5% H_2_O (*K*_A_ = 1.1 × 10^5^ m ^−1^), while its acyclic counterpart **60** has an affinity for the same anion 2.4 fold weaker in the same conditions. Computational analysis revealed that this difference is due to the presence of a rigid cavity in receptor **59**, which allows for the participation of a pyrrole NH group in the binding complex, which is not participating in the host–guest complexation for the acyclic **60**.

In this case, the insertion of rigidifying elements proved to be an efficient strategy to construct shape‐persistent receptors with higher binding affinities. Furthermore, the binding data of **58** and **60** are similar across all tested anions, suggesting that the addition of a pyrrolic hydrogen bond (**60**) compensates for the loss of the amide group (**58**). The association constants for the ether‐bridged compounds **61** and **62** proved to be similar with each other and to compound **47**, with the highest affinity for sorbate (*K*_A_ = 4.67 × 10^4^ m ^−1^ and *K*_A_ = 2.95 × 10^4^ m ^−1^ respectively). In these cases, intramolecular hydrogen bonding occurs, partially deactivating them in terms of binding. Finally, among the two acyclic derivatives **63** and **64**, the smaller **63** showed higher association constants toward all the anions tested. In fact, the bigger **64** interacts with the anion only on “one side” of the receptor, resulting in several intramolecular interactions, which make the receptor less prone to engage in the binding process. This supports the data obtained for the series of compounds **44–56**, where increasing the cavity size led to a decrease in the binding affinity. From a broader perspective, although high association affinities are achieved, receptors for carboxylates generally exhibit limited selectivity, as binding occurs exclusively at the carboxylate moiety, which remains identical across different guests.^[^ ^78^ ^]^ The association constants for compounds **44**–**64** are summarized in Table 3.

**Table 3:** Association constants (m ^−1^) for compounds **44–64** toward carboxylate guests. Unless specified, the titrations are performed via NMR in DMSO‐d_6_ + 0.5% H_2_O.

Receptor	*K*_A_ (acetate)	*K*_A_ (pivalate)	*K*_A_ (sorbate)	*K*_A_ (benzoate)	*K*_A_ (formate)	*K*_A_ (lactate)	Ref.
**44**	2.14 × 10^3^	3.39 × 10^3^	‐	1.58 × 10^4^	6.46 × 10^3^	1.94 × 10^3^	^[^ ^74^ ^]^
**45**	3.16 × 10^3^	4.90 × 10^3^	‐	851	478	251	^[^ ^75^ ^]^
**46**	2.95 × 10^4^	2.51 × 10^4^	‐	4.90 × 10^3^	3.01 × 10^3^	977	^[^ ^75^ ^]^
**47**	10^5^	8.51 × 10^4^	‐	1.48 × 10^4^	1.15 × 10^4^	2.29 × 10^3^	^[^ ^75^ ^]^
**48**	1.48 × 10^5^	7.94 × 10^4^	‐	2.95 × 10^4^	3.24 × 10^4^	2.95 × 10^3^	^[^ ^75^ ^]^
**49**	4.90 × 10^5^	4.37 × 10^5^	‐	8.12 × 10^4^	6.31 × 10^4^	1.10 × 10^4^	^[^ ^75^ ^]^
**50**	2.19 × 10^5^	1.70 × 10^5^	‐	4.37 × 10^4^	4.27 × 10^4^	4.17 × 10^3^	^[^ ^75^ ^]^
**51**	4.90 × 10^5^	6.60 × 10^5^	‐	8.91 × 10^4^	3.89 × 10^4^	1.17 × 10^4^	^[^ ^75^ ^]^
**52**	2.29 × 10^5^	1.62 × 10^5^	‐	3.80 × 10^4^	1.51 × 10^4^	4.90 × 10^3^	^[^ ^75^ ^]^
**53**	1.51 × 10^5^	2.69 × 10^5^	‐	4.17 × 10^4^	10^4^	6.03 × 10^3^	^[^ ^75^ ^]^
**54**	10^5^	2.51 × 10^5^	‐	2.57 × 10^4^	7.24 × 10^3^	4.17 × 10^3^	^[^ ^75^ ^]^
**55**	9.33 × 10^4^	2.75 × 10^5^	‐	2.14 × 10^4^	3.80 × 10^3^	5.75 × 10^3^	^[^ ^75^ ^]^
**56**	9.33 × 10^4^	3.02 × 10^5^	‐	2.10 × 10^4^	5.89 × 10^3^	3.55 × 10^3^	^[^ ^75^ ^]^
**57**	2.00 × 10^3^	2.88 × 10^3^ ^[a]^	‐	724	457	478^[a]^	^[^ ^75^ ^]^
**58**	6.61 × 10^4^	1.45 × 10^5^	‐	1.58 × 10^4^	6.46 × 10^3^	1.95 × 10^3^	^[^ ^75^ ^]^
**59**	1.15 × 10^5^	‐	7.41 × 10^4^	1.66 × 10^4^	1.91 × 10^4^	5.37 × 10^3^	^[^ ^76^ ^]^
**60**	4.47 × 10^4^	‐	3.16 × 10^4^	1.17 × 10^4^	5.01 × 10^3^	2.34 × 10^3^	^[^ ^76^ ^]^
**61**	1.29 × 10^4^	‐	2.95 × 10^4^	7.41 × 10^3^	1.32 × 10^3^	661	^[^ ^76^ ^]^
**62**	1.86 × 10^4^	‐	4.67 × 10^4^	7.41 × 10^3^	1.86 × 10^3^	550	^[^ ^76^ ^]^
**63**	7.08 × 10^4^	‐	8.71 × 10^4^	2.04 × 10^4^	5.89 × 10^3^	3.80 × 10^3^	^[^ ^76^ ^]^
**64**	3.39 × 10^3^	‐	4.68 × 10^3^	1.10 × 10^3^	661	355	^[^ ^76^ ^]^

^[a]^ Measured via UV/vis absorption titrations.

**Figure 12:**
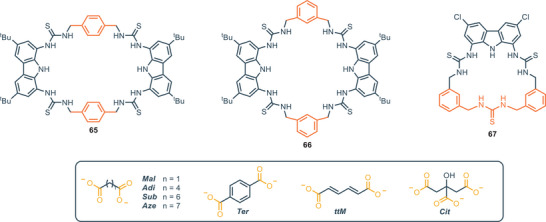
Mono and ditopic thiourea‐based macrocycles for binding of dicarboxylates.

**Figure 13:**
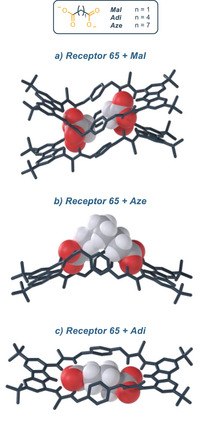
X‐ray structure of the **65·Mal** complex a), **65·Aze** complex b) and **65·Adi** complex c). Adapted from ref. [79].

In 2020, Joliffe and coworkers reported the synthesis of the tetrathiourea macrocyclic carbazoles receptors **65** and **66**, to investigate the cooperative effect of the two diaminocarbazole moieties in the binding of dicarboxylate anions (Figure 12).^[^ ^79^ ^]^ The two compounds show comparable binding affinities for a series of linear and flexible dicarboxylates (with the higher association constant for adipate, namely **Adi**). This suggests that, despite the different pore sizes of the two receptors, the macrocyles feature a certain degree of flexibility, which allows for their structural adaptation to flexible guests. When rigid dicarboxylate guests (e.g., terephthalate, namely **Ter,** and trans, trans‐muconate, namely **ttM**) are used, the difference in association constants between the two receptors becomes more pronounced: receptor **65** binds **Ter** and **ttM** with affinities that are three and two times higher, respectively, than those of receptor **66** in DMSO/H_2_O 9:1 (measured via UV/vis titrations). This can be ascribed to the effects of geometry match and mismatch, which become more pronounced as the rigidity of the guest increases. In addition, double mutant cycle analysis (DMC) provided insights into the chelate cooperativity in the binding process.^[^ ^80^ ^]^ The binding process between a ditopic guest and a ditopic host involves an initial binding event at one end of the complex (defined by the constant *K*_ini_), followed by a second binding event (characterized by *K*_intra_), that leads to the 1:1 complex. The association constant *K*_intra_ determines the chelate cooperativity. *K*_intra_ > 1 (log(*K*_intra_) > 0) reflects positive cooperativity, with the preferred formation of the 1:1 complex; *K*_intra_ < 1 (log(*K*_intra_) < 0) indicates negative cooperativity, where the second binding event is less favored, leading to the preferential formation of high‐order oligomeric species.^[^ ^79^, ^81^ ^]^ The authors observe that for small (malonate, namely **Mal**) and large (suberate, namely **Sub** and azepate, namely **Aze**) dicarboxylate anions, negative chelate cooperative effect occurs, indicating preferred oligomerization. On the other hand, the medium sized flexible dicarboxylate (**Adi**) and rigid dicarboxylate (**Ter** and **ttM**) showed moderate to strong positive cooperativity. This data reflects the differences in the association constant of the receptors varying the anion. X‐ray analysis shows that the cavity of receptor **65** is too wide to accommodate **Mal** cooperatively; the malonate forms a 2:2 complex, where two molecules of **65** are held together by hydrogen bonding interaction forming a sandwich‐like complex (Figure 13a). On the other hand, the X‐ray structure of **65** with **Aze** shows that the anion does not completely fit in the cavity, with the alkyl chain remaining outside of the cavity (Figure 13b). **Adi** represents a striking balance in terms of dimension, since it is perfectly encapsulated in the macrocycle itself (Figure 13c). This evidence fully supports both its higher association constant and its positive chelate cooperativity. A few years later, the same group reported macrocycle **67**, which does not feature a ditopic design. Compound **67** displays an intense and selective fluorescence response toward citrate (**Cit**), and it was applied as a fluorescence turn‐on sensor for detection of the citrate in living cells.^[^ ^82^ ^]^

### 2.2 1,8‐Disulfonamide–Carbazole Platform

**Figure 14:**
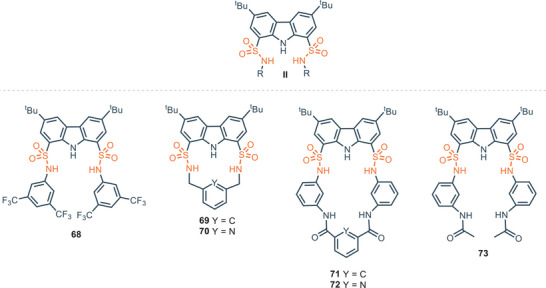
1,8‐disulfonamide‐carbazole as acyclic and cyclic anion binding receptors.

A different family of carbazole receptors is based on 1,8‐disulfonamide–carbazole scaffold **II**, featuring the heterocyclic core functionalized with sulfonamide moieties in position 1 and 8 (Figure 14). The synthetic protocol involves generally the introduction of *t*‐Bu groups at positions 3 and 6 of carbazole, followed by sulfonation at positions 1 and 8. The reaction with PCl_5_ provide the 1,8‐disulfonylchloride precursor, which furnishes 1,8‐disulfonamidecarbazole‐based receptors reacting with the desired amines.^[^ ^83^ ^]^ One important example of receptors based on this scaffold was developed by Alcazar and co‐workers.^[^ ^83^ ^]^ As shown in Figure 14, receptor **68** displays three NH donor sites, two from the sulfonamide moiety and one from the carbazole skeleton. **68** can bind halides with high affinity, especially chloride which is bound with an association constant of 7.9 × 10^6^ m ^−1^ in CDCl_3_. This anion tends to form weak supramolecular interactions and in general is challenging to selectively bound it when other anions are present in solution. Despite these characteristics, chloride plays a crucial role in biological systems because it is the only inorganic anion involved in transmembrane gradients in cells.^[^ ^84^ ^]^ As a comparison with amide, urea or thiourea receptors **1**, **2** (Figure 2) and **30** (Figure 8), the substantial difference with 1,8‐disulfonamide–carbazole is that NH sites, beyond carbazole‐NH ones, are not directly linked to the carbazole backbone, but to the SO_2_ moieties. In 1,8‐disulfonamide–carbazole receptors sulfonamide‐NH sites are not fixed in a rigid position; hence they experiment greater mobility and adaptability toward anions. As an example, receptor **2** binds chloride asymmetrically when this anion is positioned in the carbazole cleft; amide‐NH─Cl^−^ bond lengths are about 2.94 and 2.60 Å in Et_2_O and the strongest interaction occurs with the NH of the carbazole;^[^ ^25^ ^]^ while, in the case of **68** crystal structures reveal the existence of two symmetrical H‐bonds between sulfonamide‐NHs and chloride. The more flexible NH sulfonamide groups have the possibility to establish a closer interaction with the anion, while the carbazole NH seems to be too far to be involved in binding.^[^ ^83^ ^]^ This aspect probably contributes to the higher affinity of **68** toward chloride in DMSO, combined with 1) the presence of *t*‐butyl substituents on the carbazole scaffold, which help the receptor to adopt a favorable conformation and 2) the presence of strong electron‐withdrawing groups like 3,5‐bis(trifluoromethyl) groups.,^[^ ^85^ ^]^ A comparison between urea, thiourea and sulfonamide‐based receptors in terms of binding affinity could be performed considering the affinity of **30**, **32**, and **68** toward chloride in DMSO. Each receptor bears 3,5‐bis(trifluoromethyl) substituents on the aryl appendages and the affinity increases following the order thiourea‐**32** < sulfonamide‐**68** < urea‐**30** (see Tables 2 and 4). More recently, a series of carbazole sulfonamide‐based macrocycles were prepared by Bao and colleagues.^[^ ^86^ ^]^ **69** and **70** are constituted by 1,8‐disulfonamidecarbazole platform and 1,3‐xylyl or 2,6‐lutidinyl linker (Figure 14). High affinity is shown toward different anions, in particular **69** displays strong and selective binding toward fluoride, with association constants up to 5 × 10^4^ m ^−1^ in acetonitrile. Nevertheless, the affinity is dramatically lower in polar solvents, e.g., in DMSO it shows a *K*_A_ = 147 m ^−1^. This behavior can be ascribed to a strong interaction of polar solvent with the guest and host binding site. To overcome this issue, receptors **71**–**73** were designed with additional NH donor groups in the structure to maximize the affinity over the anions in strongly polar media.^[^ ^87^ ^]^ Receptors **71**and **72** are among the first examples of carbazole bis‐sulfonamide‐bis‐amide macrocycles developed for anion recognition and they are particularly sensitive toward F^−^, CH_3_COO^−^, PhCOO^−^, and H_2_PO_4_^−^_._ Macrocycles **71** and **72** were compared with the acyclic analogue **73** to evaluate the macrocyclic effect. As expected, the affinity toward anions drastically drops considering receptor **73**. Nevertheless, **71** and **72** showed a unique behavior toward chloride highlighted by a pronounced downfield shift of amide NH groups while no changes are detected for carbazole and sulfonamide NH moieties by ^1^H‐NMR analysis.

**Table 4:** Association constants (m ^−1^) for compounds **68**–**74** toward different guests. Unless specified, the titrations are performed via NMR.

Receptor	*K*_A_ (acetate)	K_A_ (benzoate)	*K*_A_ (Cl^−^)	*K*_A_ (F^−^)	*K*_A_ (Br^−^)	*K*_A_ (H_2_PO_4_^−^)	Ref.
**68** ^[^ ^a^ ^]^	^[^ ^b^ ^]^	‐	7.9 × 10^6^^[^ ^c^ ^]^ 210 (DMSO‐d_6_)	2.2 × 10^4^	1.1 × 10^6^^[^ ^d^ ^]^ 25 (DMSO‐d_6_)	^[^ ^b^ ^]^	^[^ ^83^ ^]^
**69** ^[^ ^e^ ^]^	279	1.01 × 10^3^	91	5.09 × 10^4^^[^ ^f^ ^]^	24	4.92 × 10^3^	^[^ ^86^ ^]^
**70** ^[^ ^e^ ^]^	940	2.01 × 10^3^	355	1.29 × 10^4^	<10	2.43 × 10^3^	^[^ ^86^ ^]^
**71** ^[^ ^g^ ^]^	7.30 × 10^3^	6.80 × 10^3^	30	1.53 × 10^4^^[^ ^f^ ^]^	n.d.	611	^[^ ^87^ ^]^
**72** ^[^ ^g^ ^]^	1.00 × 10^4^	6.80 × 10^3^	165	1.09 × 10^4^^[^ ^f^ ^]^	n.d.	688	^[^ ^87^ ^]^
**73** ^[^ ^g^ ^]^	2.70 × 10^3^	2.40 × 10^3^	17	1.08 × 10^4^^[^ ^f^ ^]^	n.d.	218	^[^ ^87^ ^]^
**74** ^[^ ^e^ ^]^	5.10 × 10^4^	‐	5.90 × 10^3^	1.61 × 10^4^	2.90 × 10^3^	2.75 × 10^4^	^[^ ^88^ ^]^

^[a]^ In CDCl_3_.

^[b]^ Data could not be fitted to a 1: 1 or 1: 2 binding stoichiometry.

^[c]^ Measured via competitive fluorescence titration with bromide.

^[d]^ Measured via fluorescence.

^[e]^ In CD_3_CN.

^[f]^ Determined via UV–vis titration experiments.

^[g]^ In DMSO‐d_6_.

**Figure 15:**
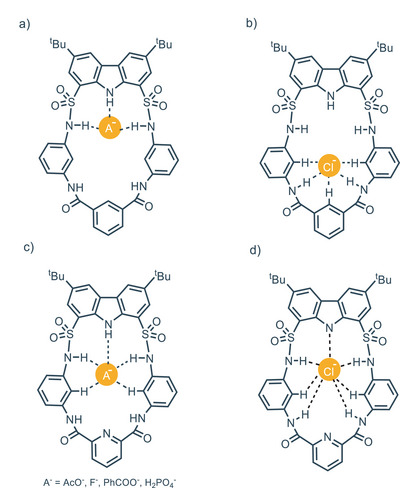
a) receptor **71** involved in the binding of different anion A^−^; b) receptor **71** involved in the binding of chloride; c) receptor **72** involved in the binding of different anion A^−^; d) receptor **72** involved in the binding of chloride. Adapted from ref. [87].

**Figure 16:**
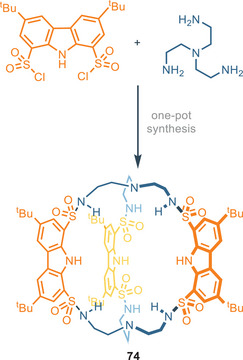
Three unit‐carbazole cryptand developed by Bao et al. Adapted from ref. [88].

On the other hand, chemical shift changes of carbazole and sulfonamide protons occur when CH_3_COO^−^, PhCOO^−^, F^−^, and H_2_PO_4_^−^ are present. This observation suggests that receptor **71** has two distinct binding sites, one pocket constituted by carbazole and sulfonamide NHs that binds CH_3_COO^−^, PhCOO^−^, F^−^, and H_2_PO_4_^−^ (Figure 15a); the other one constituted by two amide NHs sites and three directed aromatic CH protons which can accommodate Cl^−^ (Figure 15b). Regarding **72**, because of a hydrogen donor site in the amide pocket replaced with a nitrogen in the aromatic ring, chloride interacts much more with the carbazole/sulfonamide pocket (Figure 15d); in the case of F^−^, CH_3_COO^−^, PhCOO^−^, and H_2_PO_4_^−^, the model proposed shows also the involvement of aromatic CH (Figure 15c). In 2023, Bao and colleagues presented a novel carbazole‐1,8‐disulfonamide cryptand‐like for anion recognition.^[^ ^88^ ^]^ Cryptand **74** comprises three 1,8‐disulfonamidecarbazole units held together by two tris(2‐aminoethyl)amine (TREN) moieties as linkers. **74** is synthesized in a straightforward manner through a one‐step [2 + 3] condensation (Figure 16).

The binding affinity was measured toward different monovalent anions, including AcO^−^, H_2_SO_4_^−^, F^−^, and Cl^−^. Cryptand **74** exhibits strong binding principally toward AcO^−^, F^−^, and H_2_PO_4_^−^ even in polar solvents like DMSO, with association constants up to 8 × 10^3^ m ^−1^ for 1:1 complex. The scarce influence of solvent polarity on association constants is a consequence of the reduced solvation shells in the binding cavity of these hosts. Specifically, **74** shows a clear preference for AcO^−^ with a *K*_A_ of 5.1 × 10^4^ m ^−1^ in a less polar solvent like CH_3_CN, assuming that all the available NHs bind acetate anion both in DMSO and CH_3_CN. The association constants for compounds **68**–**74** are summarized in Table 4.

### 2.3 Indolo[2,3‐α]Carbazole Platform

**Figure 17:**
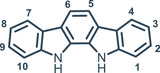
Numbered scaffold of indolo[2,3‐α]carbazole.

Starting from the mid 2000s, indolocarbazole has been used as a platform for anion recognition, displaying strong binding affinity and selectivity.^[^ ^89^, ^90^, ^91^ ^]^ Indolocarbazoles are a family of different isomers characterized by pentacyclic ring system of fused indole and carbazole moieties. In particular the indolo[2,3‐*α*]carbazole isomer is a suitable platform for the development of synthetic anion receptors, even though the synthesis of 1,10‐substituted‐indolocarbazoles is still limited. The double Fischer indole synthesis from pre‐functionalized precursors represents the general synthetic route to access the indolo[2,3‐*α*]carbazole scaffold, however, this strategy fails with the introduction of strong electron‐withdrawing groups. For this reason, unlike carbazoles, 1,10‐diamino‐indolocarbazoles have never been reported, because of the difficult access to 1,10‐dinitro‐indolocarbazole precursor. The skeleton of indolo[2,3‐α]carbazole features characteristics analogues to carbazole, like high preorganization and rigidity. Nevertheless, the indolocarbazole scaffold provides an additional well‐orientated NH group as hydrogen bond donor sites for the recognition of anions (Figure 17).

**Figure 18:**
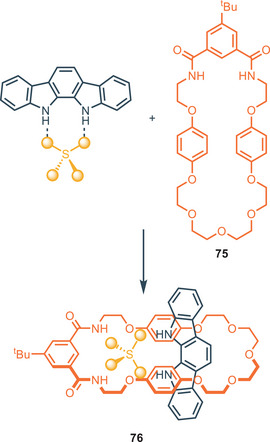
Sulfate anion templating of rotaxane **76** in the presence of indolo[2,3‐α]carbazole. Adapted from ref. [92].

The Beer group made a pioneering contribution in this field using indolo[2,3‐α]carbazole as an axle in templated rotaxanes formation in the presence of different anions (Figure 18). Rotaxane **76** is obtained between indolocarbazole and compound **75** in the presence of SO_4_^−^ in CH_3_CN.^[^ ^92^ ^]^ Different relevant works were conducted by the same group in this regard during the following years.^[^ ^93^, ^94^, ^95^ ^]^

**Figure 19:**
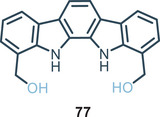
Di(hydroxymethyl)indolocarbazole **77** synthetized by Molina and co‐workers.

Despite some indolocarbazole isomers could be naturally isolated, the double Fischer indolization is the synthetic route generally used for their preparation.^[^ ^96^ ^]^ The arch‐shaped cavity makes indolo[2,3‐*α*]carbazole optimal toward “Y‐shaped” anions like carboxylates. Indeed, unsubstituted indolo[2,3‐*α*]carbazole (Figure 17) binds benzoate with an association constant of *K*_A_ = 1 × 10^5^ m ^−1^ in acetonitrile. In this regard, Molina and co‐workers developed different1,10‐disubstituted indolo[2,3‐*α*]carbazoles sensitive toward “Y‐shaped” anions.^[^ ^96^ ^]^ In detail Figure 19 shows receptor **77** which binds strongly benzoate with an association constant higher than *K*_A _= 10^7^ m ^−1^ via NMR in acetonitrile (Table 5) thanks to the presence of two additional OH donor sites.

**Table 5:** Association constants (m ^−1^) for compounds **77–84**. The titrations are performed via NMR in DMSO‐d_6_ + 0.5% H_2_O toward chloride and in DMSO‐d_6_ + 10% CD_2_Cl_2_ toward nitrate.

Receptor	K_A_ (Cl^−^)	K_A_(NO_3_^−^)	K_A_(benzoate)	Ref.
**77**	‐	‐	>10^7^^[^ ^a^ ^]^	^[^ ^96^ ^]^
**78**	32	26	‐	^[^ ^101^ ^]^
**79**	36	30	‐	^[^ ^101^ ^]^
**80**	216	141	‐	^[^ ^101^ ^]^
**81**	5.84 × 10^3^	1.30 × 10^4^	‐	^[^ ^101^ ^]^
**82**	1.17 × 10^4^	2.30 × 10^4^	‐	^[^ ^101^ ^]^
**83**	K_1_ = 68, K*_2 _* = 16	78	‐	^[^ ^101^ ^]^
**84**	K*_1_* = 31, K*_2_* = 9	31	‐	^[^ ^101^ ^]^

^[a]^ In acetonitrile via fluorescence analysis.

**Figure 20:**
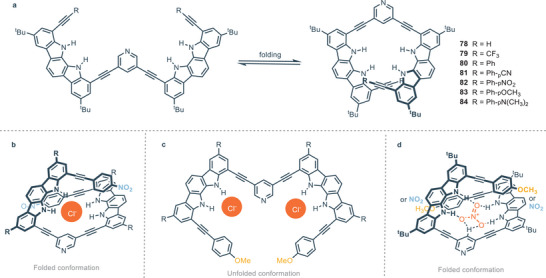
a) Foldamers based on indolo[2,3‐α]carbazole and b–d) related conformations based on electron‐withdrawing or electron‐donating aryl‐appendages in the presence of chloride or nitrate anions. Adapted from ref. [101].

A comprehensive and extended review about the origin, synthesis, properties and applications of indolocarbazole has been recently published;^[^ ^97^ ^]^ hence, herein only recent developments in this field will be discussed. Indolo[2,3‐*α*]carbazole scaffold shows significant impact as a building block for the rational construction of synthetic helical foldamers. The helical conformation of proteins and enzymes provides the formation of a cavity that offers hydrogen binding sites for the interaction and accommodation of guests. In this regard it is a great challenge to design and construct synthetic foldamers that can mimic the behavior of biological receptors. In 2008, Jeong and colleagues presented synthetic water‐soluble foldamers based on indolo[2,3‐a]carbazole units tied together by ethynyl linkers. These foldamers can bind halides thanks to the formation, upon folding, of tubular cavities.^[^ ^98^ ^]^ During the following years, Jeong developed foldamers with related structures inserting an heteroaromatic spacer that acts like a fulcrum and different appendages.^[^ ^99^, ^100^ ^]^ The authors demonstrated that it is possible to tune the binding affinity of synthetic foldamers by intra‐receptor π‐staking interactions provided by aryl‐appendages.^[^ ^101^ ^]^ These foldamers consist of two indolo[2,3‐*α*]carbazoles equipped with different appendages. The two indolocarbazole units are tied together by ethynyl linkers spaced by pyridine which acts as a fulcrum (Figure 20a). Receptors **78** and **79** without any aryl‐appendages provide association constants with 3 orders of magnitude lower than receptors equipped with aryl‐appendages (*K*_A_ **78** = 32 m ^−1^, *K*_A_ **78** = 36 m ^−1^, *K*_A_ **82** = 1.17 × 10^4^ m ^−1^) in DMSO. The helical folding allows face‐to‐face π‐stacking interactions between the aryl‐appendages and the indolocarbazole backbone. The intra‐receptor interactions do not directly involve the guest but increase binding affinity through the stabilization of the entire folding structure. In the case of electron‐withdrawing groups on the aryl‐appendages, receptors **81** and **82** assume a completely folded conformation, leading to a 1:1 complex with chloride anion (Figure 20b). On the other hand, the presence of electron‐donating groups on aryl‐appendages leads to a completely different scenario for the binding of chloride. In particular, the conformation of **83** and **84** is extended and X‐ray structures show complexes 1:2, i.e., one chloride atom for each indolocarbazole unit (Figure 20c). This is consistent with intra‐receptor π‐staking interactions which are favored by electron‐withdrawing substituents because of attractive electrostatic interactions. The bigger trigonal planar nitrate anion maximizes hydrogen bonding interactions in the helical conformation, forming 1:1 complex with foldamers, regardless of the nature of the aryl‐substituent (Figure 20d). The same group recently developed similar foldamers, with a 1,8‐naphthyridine moiety as the fulcrum between indolecarbazole units. In this way, the cavity formed by helical folding is wider and it is possible to bind carbohydrates derivatives, as described in Section 3. The association constants for compounds **77**–**84** are summarized in Table 5.

### 2.4 Miscellaneous Carbazole Platforms

**Figure 21:**
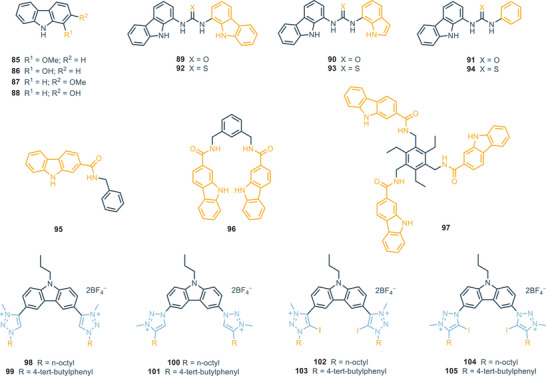
Miscellaneous carbazole‐based receptors.

This section will address the binding properties and potential applications of miscellaneous carbazole‐based receptors. Notably, most receptors depicted in Figure 21 lack similarities in functional group composition, unlike the receptors discussed in the previous paragraphs, which were grouped based on their common characteristics related to a specific functional group.

Receptors **85**–**88** have been extensively studied by Menéndez and coworkers due to their enhanced fluorescence properties compared to carbazole.^[^ ^102^ ^]^ These receptors have been explored as potential fluorescent sensors in acetone for various anions, including F^−^, Cl^−^, Br^−^, CN^−^, AcO^−^, and OH^−^. Among them, receptor **86** exhibited the highest sensitivity with notable selectivity toward the halides F^−^ (*K*_A_ = 9.64 × 10^4^ m ^−1^) and Cl^−^ (*K*_A_ = 1.55 × 10^5^ m ^−1^). This sensitivity can be attributed to the spherical geometry of these anions. In fact, F⁻ and Cl⁻ exhibit a higher efficiency in displacing solvent molecules. This displacement facilitates direct interaction of the anions with the receptor, leading to a significant increase in fluorescence intensity.^[^ ^102^ ^]^ This can be also attributed to the fact that F^−^ and Cl^−^ induce a more rigid conformation in the carbazole structure, enhancing its fluorescence properties.^[^ ^102^ ^]^

Urea‐based receptors **89**–**91** exhibit a preferential binding affinity for oxoanions in DMSO‐d_6_ + 0.5% H_2_O. Notably, all three receptors demonstrate significant affinity toward acetate and bicarbonate with *K*_A_ > 10^4^ m ^−1^.^[^ ^103^ ^]^ The corresponding thiourea‐based receptors **92**–**94** unexpectedly display a reduced binding affinity (e.g., *K*_A_ > 10^3^ m ^−1^ for acetate), despite the increased acidity of the thiourea group relative to the urea group. This behavior could be attributed to conformational effects, as the larger size of sulfur compared to oxygen may induce a “twisted” geometry.^[^ ^103^ ^]^ Table 6 shows the association constant for receptors **89**–**94**.

**Table 6:** Association constants (m ^−1^) for receptors **89**–**94**. The titrations are performed via NMR in DMSO‐d_6_ + 0.5% H_2_O.^[^ ^103^ ^]^

Receptor	K_A_ (acetate)	K_A_ (benzoate)	K_A_ (H_2_PO_4_^−^)	K_A_ (Cl^−^)	K_A_(HCO_3_^−^)
**89**	>10^4^	5.67 × 10^3^	n.d.	102	>10^4^
**90**	>10^4^	5.88 × 10^3^	n.d.	139	>10^4^
**91**	>10^4^	3.42 × 10^3^	6.14 × 10^3^	85	>10^4^
**92**	223	658	687	15	n.d.
**93**	1.80 × 10^3^	675	1.34 × 10^3^	17	n.d.
**94**	1.78 × 10^3^	870	n.d.	23	n.d.

n.d. = the association constant could not be determined

Receptors **95**–**97** were synthesized and studied as ligands for a series of anions. Association constant studies were conducted for acetate, H_2_PO_4_^−^ and HP_2_O_7_^−3^.^[^ ^104^ ^]^

It was observed that, in general, receptor **95** exhibits weaker binding to anions in DMSO‐d_6_ compared to **96** and **97**. Indeed, receptor **96** demonstrates a notable association constant toward HP_2_O_7_^−3^ (K_A_ > 10^3^ m ^−1^), whereas receptor **97** displays such a high affinity for HP_2_O_7_^−3^ that its association constant could not be determined in DMSO‐d_6_.

Only the addition of 5% water to DMSO‐d_6_, creating a more competitive environment for anion binding, allowed the calculation of the association constant for receptor **97** with HP_2_O_7_^−3^ (*K*_A _= 145 m ^−1^).^[^ ^104^ ^]^ Table 7 shows the association constants for receptors **95**–**97**.

**Table 7:** Association constants (m ^−1^) for receptors **95**–**97**. The titrations are performed via NMR in DMSO‐d_6_. ^[^ ^104^ ^]^

Receptor	K_A_ (acetate)	K_A_(H_2_PO_4_^−^)	K_A_(P_2_O_7_4^−^)
**95**	55	14	n.d.
**96**	65	20	>10^4^
**97**	165	440	n.d.

n.d. = The association constant could not be determined

Carbazole‐based cationic receptors featuring *N*‐alkylated and disubstituted triazolium groups (**98**–**105**) have been investigated for their anion‐binding properties in CD_3_CN.^[^ ^105^ ^]^ Binding affinities were evaluated toward halide anions and selected oxoanions, such as nitrate and sulfate. Generally, no significant difference in binding efficiency was observed between receptors with a carbazole‐triazolium linkage of the C─C type (**98**, **99**, **102**, **103**) and those with a C─N linkage (**100**, **101**, **104**, **105**). However, differences in anion‐binding behavior became more pronounced when comparing receptors with alkyl versus aromatic pendants. Receptors with aromatic substituents (**99**, **101**, **103**, **105**) exhibited stronger anion‐binding affinities than those with alkyl substituents (**98**, **100**, **102**, **104**).^[^ ^105^ ^]^ Substantial differences in halide binding were observed between receptors functionalized with iodine (**102**–**105**) and those without iodine functionalization (**98**–**101**).

Iodine‐functionalized receptors, also referred to as XB receptors, demonstrated enhanced halide ion interactions through halogen bonding, in contrast to the HB receptors, which rely solely on hydrogen bonding.^[^ ^105^ ^]^ Regarding sulfate binding, receptors **99** and **101** (bearing aromatic substituents and lacking iodine functionalization) exhibited the strongest affinities, even in competitive solvent mixtures such as DMSO‐d_6_ with 10% D_2_O. This behavior is primarily driven by the Coulombic attraction between the dicationic receptor and the dianionic guest.^[^ ^105^ ^]^

**Figure 22:**
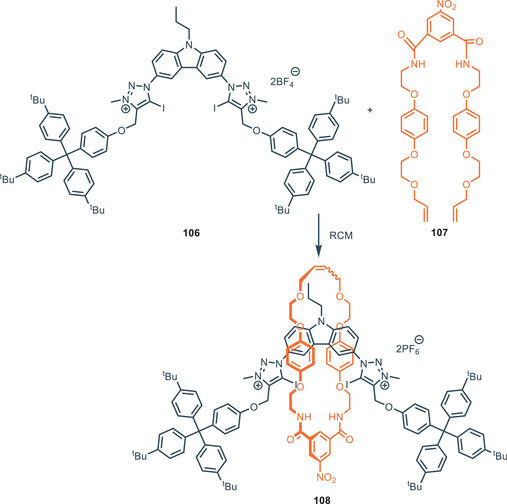
Synthesis of rotaxane **108**·2PF_6_ via RCM.

In this context, to exploit the efficiency of XB‐type receptors toward chloride and bromide, coupled with an isophthalamide‐based macrocycle, rotaxane **108** was synthesized. The synthesis was achieved via Grubbs olefin metathesis of **107** in combination with the XB bis‐iodotriazolium carbazole axle component **106** (Figure 22). Following purification, the rotaxane was subjected to anion exchange with aqueous NH_4_PF_6_, affording **108**·2PF_6_. The anion‐binding properties of rotaxane **106**·2PF_6_ were examined using ^1^H‐NMR in a 45:45:10 mixture of CDCl_3_/CD_3_OD/D_2_O. The results demonstrated a pronounced affinity for halides, in particular for bromide ions, over oxoanions. Notably, this behavior differs from that of simpler aryl‐based XB receptors (**103** and **105**), which exhibit a higher binding affinity for chloride ions.^[^ ^105^ ^]^ Table 8 shows the association constants for receptors **98**–**105**.

**Table 8:** Association constants (m ^−1^) for receptors **98**–**105**. The titrations are performed via NMR in DMSO‐d_6._^[^ ^105^ ^]^

Receptor	K_A_ (Cl^−^)	K_A_(Br^−^)	K_A_(I^−^)	K_A_ (NO_3_^−^)	K_A_ (SO_4_^−2^)^[^ ^b^ ^]^
**98**	141	200	161	121	341
**99**	430	337	214	295	1.54 × 10^3^
**100**	214	274	233	244	282
**101**	240	287	310	213	1.03 × 10^3^
**102**	217^[^ ^a^ ^]^	567	99	‐	348
**103**	332^[^ ^a^ ^]^	893	254	‐	493
**104**	341^[^ ^a^ ^]^	367	99	‐	363
**105**	303^[^ ^a^ ^]^	900	222	‐	652

^[a]^ Titrations conducted in 10% D_2_O/DMSO‐d_6_.

^[b]^ Titration conducted in 10% D_2_O/DMSO‐d_6_. n.d. = the association constant could not be determined

## 3 Carbazole‐Based Receptors for Biologically Relevant Organic Molecules

**Figure 23:**
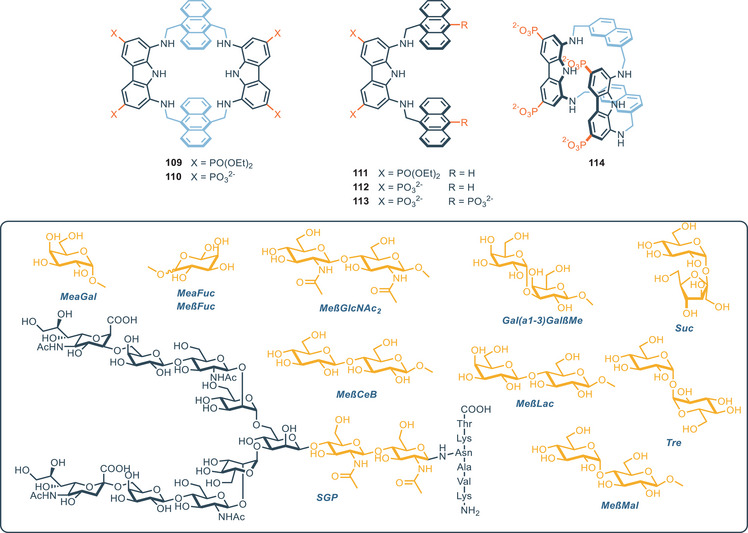
1,8‐diaminocarbazole‐based phosphonate receptors for carbohydrates and xanthines.

In addition to anions, carbazole‐based receptors have also been applied for the recognition of organic molecules. Essential biological compounds, like xanthines,^[^ ^106^, ^107^, ^108^, ^109^, ^110^ ^]^ carbohydrates,^[^ ^111^, ^112^, ^113^, ^114^, ^115^ ^]^ and amino acids,^[^ ^116^, ^117^ ^]^ play a key role in various physiological processes, making them important targets for selective synthetic receptors.^[^ ^118^ ^]^ In 2018, Francesconi and coworkers developed the first 1,8‐diaminocarbazole‐based synthetic receptor for the molecular recognition of saccharides in water (Figure 23).^[^ ^119^ ^]^ The macrocyclic compound **110** displays anthracene moieties linking two diaminocarbazole units, thus defining a shape‐persistent hydrophobic cavity capable to locate monosaccharides. The carbazoles are also equipped with phosphonate hydrophilic groups, which allows for solubility and recognition in water. The affinities, assessed through NMR and ITC titrations, were determined using intrinsic median binding concentration (BC^0^_50_) parameter. BC^0^_50_ describes the global affinity of a guest for a host when multiple species at different stoichiometry are present.^[^ ^120^ ^]^ It is also important to consider that the carbohydrates used in the binding experiments are methylated at the anomeric oxygen to prevent mutarotation. Receptor **110** proved to bind Meα‐fucose (MeαFuc) in water with unprecedently reported affinity in the micromolar range (BC^0^_50_ = 0.36 ± 0.09 mm) and high selectivity with respect to the corresponding β anomer. Molecular modeling, together with NOESY NMR analysis, revealed that 5 of the 6 NH groups participate in hydrogen bonding interaction with the hydroxyl groups of the saccharide, while the axial protons are involved in CH‐π interactions with the anthracene rings. Binding properties of compound **110** were also explored toward disaccharides in water.^[^ ^121^ ^]^ The receptor proved to bind several methyl‐β‐glycosides of glucose‐containing 1–4 disaccharides such as Meβ‐maltose (MeβMal), Meβ‐cellobiose (MeβCel) and Meβ‐lactose (βLac) with good affinity, although with no selectivity among them, while it did not recognize 1–1′ disaccharides such as sucralose (Suc) and trealose (Tre). Moreover, the receptor is able to distinguish between MeβCel and its amino‐acetylated analog Meβ‐*N*,*N*′‐diacetylchitobiose (MeβGlcNAc_2_), which is not bound at all. The lack of selectivity among most of the glucose‐containing disaccharides was attributed to the small degree of adaptation of the macrocycle **110**, which is unable to fit the entire disaccharide. This results in the exclusive binding of the glucose unit of the disaccharides, leading to loss in selectivity. The tweezer‐shaped acyclic **112** was reported by the same group in 2021, with the idea that a more flexible design could be beneficial for binding of disaccharide with higher affinity and selectivity.^[^ ^122^ ^]^ Indeed, compound **112** proved to be effective in binding of all‐equatorial disaccharides in water, showing marked affinity for MeβGlcNAc_2_, with the highest value reported in literature so far (BC^0^_50_ = 160 ± 10 µm). On the other hand, monosaccharides were not bound at all, as well as 1–1′ disaccharides. Compound **112** is characterized by strong formation of dimers in solution, due to the π‐stacking interaction of the anthracene moieties. To overcome this issue, the tetraphosphonate derivative **113** was prepared.^[^ ^123^ ^]^

**Figure 24:**
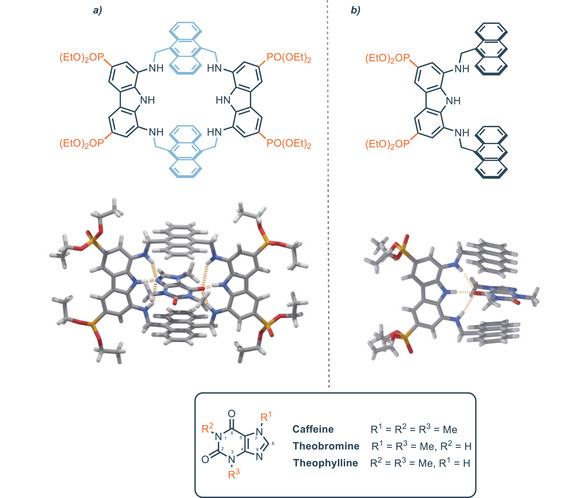
Proposed binding modes of compounds **109** and **111** toward caffeine. a) Up: compound **109**; Down: graphical representation of the minimum energy conformation of the **109·**caffeine complex. b) Up: compound **111**; Down: X‐ray structure of the **111·**caffeine complex. Adapted from ref. [125].

This compound features another phosphonate moiety on each extended aromatic platform, reducing the dimerization of the receptor, thus allowing for the structural characterization of the binding mode, especially for complex oligosaccharides. In fact, compound **113** was able to recognize the sialylglycopeptide **SGP** selectively interacting with the βGalNAc_2_ core, with an affinity comparable to that of natural lectins (BC^0^_50 _= 170 ± 50 µm determined via ITC titrations). The macrocyclic architecture **110** and the tweezer‐shaped features of **112** were combined by the same group in 2024, designing derivative **114**.^[^ ^124^ ^]^ This compound is a flexible macrocycle possessing a tweezer‐shaped‐like structure. In fact, compound **114** features two diaminocarbazole moieties bridged by a naphthalene ring through methylene groups. This receptor proved to effectively bind both MeαGal and Gal(α1–3)GalβMe (respectively, BC^0^_50_ = 1.91 ± 1.34 mm for the monosaccharide and 3.97 ± 0.54 mm for the disaccharide). The corresponding ethyl phosphonate analogs of **110** and **112**, namely **109** and **111**, were also employed as receptors for xanthines in CDCl_3_.^[^ ^125^ ^]^ Compound **109** showed marked affinity for caffeine (BC^0^_50_ = 9.3 ± 0.9 µm), although with poor selectivity, since the binding of theophylline takes place with a similar affinity. Molecular modeling revealed that the binding takes place between the two diaminocarbazole moieties and the O‐6 and the N‐9 positions of the xanthine core (Figure 24a). The flexible tweezer‐shaped **111**, on the other hand, was able to bind caffeine with a smaller affinity (BC^0^_50_ = 26.2 ± 1.6 µm) but with a marked selectivity versus other xanthines, since the binding of theophylline and theobromine occurs with markedly lower affinity (four‐ and sixfold smaller). Analysis of the single crystal of complex between **111** and caffeine reveals that CH–π interactions between **111** and the methyl groups on N−1 and N−7 play a crucial role in the binding process, along with hydrogen bonding interactions between O−6 and the diaminocarbazole unit and π‐stacking interaction between the xanthine and the anthracene core (Figure 24b).

The lack of one methyl group in theophylline and theobromine, indeed, accounts for the smaller association constant and therefore for the higher selectivity of **111** toward caffeine. Also the flexibility of the host plays a role in the binding affinity. Sulfonated analogues of receptors **112** were also employed in the selective recognition of caffeine in the aqueous media with high affinity.^[^ ^126^ ^]^ The association constants of compounds **109** and **111** toward xanthines are summarized in Table 9, while the binding data for compounds **110** and **112**–**114** are summarized in Table 10.

**Table 9:** BC_0_^50^ (mm) for compounds **109** and **111** toward xanthines. The titrations are performed via NMR in CDCl_3_

Receptor	BC_0_^50^ (Caffeine)	BC_0_^50^ (Theophylline)	BC_0_^50^ (Theobromine)	Ref.
**109**	9.3 ± 0.9	13.8 ± 4.0	‐	^[^ ^125^ ^]^
**111**	26.2 ± 1.6	118 ± 6.7	154 ± 7.2	^[^ ^125^ ^]^

**Table 10:** BC_0_^50^ (mm) for compounds **110**, **112**, **113** and **114**. The titrations are performed via NMR in D_2_O unless otherwise specified.

Receptor	BC_0_^50^ (MeαGal)	BC_0_^50^ (MeαFuc)	BC_0_^50^ (MeβFuc)	BC_0_^50^ (MeβGlcNAc_2_)	BC_0_^50^ (Galα(1,3)GalβMe)	BC_0_^50^ (MeβLac)	BC_0_^50^ (MeβCeB)	BC_0_^50^ (MeβMal)	BC_0_^50^ (SGP)	Ref.
**110**	1.19 ± 0.20	0.65 ± 0.11	20.6 ± 5.0	n.d.	—	1.43 ± 0.05	1.15 ± 0.04	1.06 ± 0.07	—	^[^ ^119^, ^121^ ^]^
**112**	—	—	—	0.16 ± 0.01	—	30.8 ± 4.7	0.94 ± 0.1	31.0 ± 4.4	—	^[^ ^121^, ^122^ ^]^
**113**	—	—	—	0.27 ± 0.01	—	9.01 ± 016	1.04 ± 0.06	11.9 ± 0.3	0.17 ± 0.05^[^ ^a^ ^]^	^[^ ^123^ ^]^
**114**	1.91 ± 0.20	27.8 ± 4.33	—	—	3.97 ± 0.54	—	—	—	—	^[^ ^124^ ^]^

^[a]^ Determined via ITC titration.

**Figure 25:**
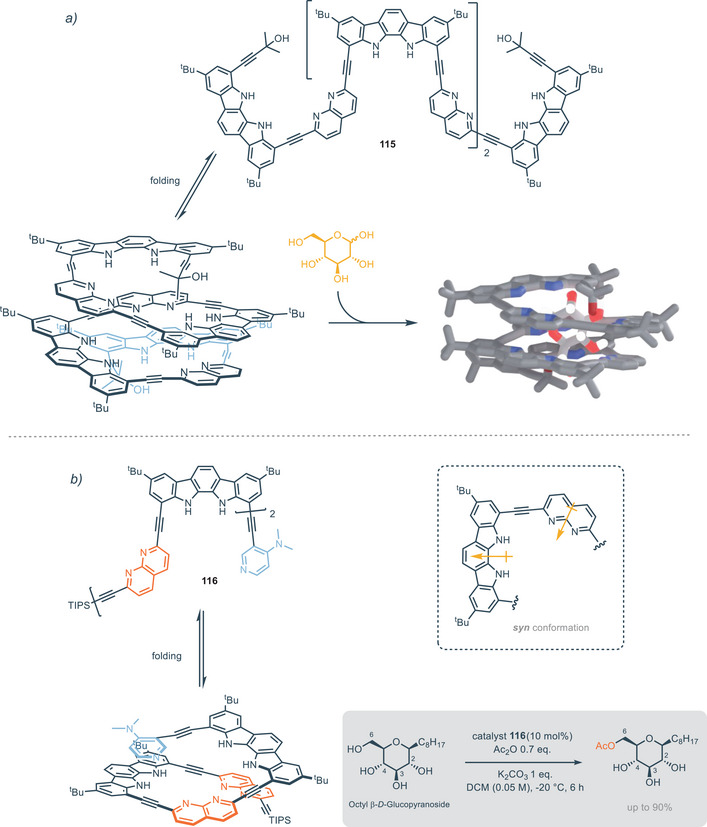
a) Molecular structure of foldamer **115** and its folding in the presence of glucose. Adapted from ref. [127]; b) *syn* conformation of foldamer **116** which acts as a catalyst for the highly regioselective acetylation of monosaccharides. Adapted from ref. [128].

**Figure 26:**
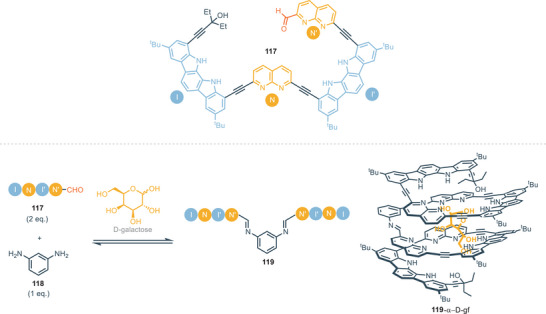
Molecular structure of monomeric foldamer **117** and diimine **119** assembly in the presence of the guest d‐galactose. The complex **119**‐α‐d‐gf is formed only with one of the d‐galactose isomers: α‐d‐galactofuranose. Consequently, the equilibrium shifts completely converting all the isomers into α‐d‐gf. Adapted from ref. [129].

Carbazole‐based foldamers are capable of interacting also with carbohydrates. In particular, aromatic foldamers constituted by repeating units of indolo[2,3‐α]carbazole are able to incapsulate monosaccharides. Hybrid aromatic foldamers can exhibit extremely high affinity and strong selectivity toward organic guests because the shape and the size of the internal cavity generated by the folding can be modulated by modifying different features in the structure of the foldamer, such as the type and number of the repeated subunits and the type and the length of the spacer that holds the subunits together. Thanks to the presence of π‐staking interactions, the helical conformation creates a cavity that is optimal for binding monosaccharides. **115** is a stable helical indolocarbazole − naphthyridine foldamer with tertiary hydroxyl group on the appendages (Figure 25a). **115** can incapsulate glucose and galactose with association constants of 9.6 × 10^4^ m ^−1^ and 1 × 10^4^ m ^−1^, respectively.^[^ ^127^ ^]^ In less strongly polar solvents, like dichloromethane and toluene, the syn‐conformation is the more stable one due to the instauration of dipole–dipole interactions. The tubular cavity created by the folding is hydrophilic, with all the NH hydrogen donor sites facing the inside of the cavity. Nevertheless, the authors demonstrate that the structure is stabilized by the presence of water molecules that form hydrogen‐bonding network with the NH and N atoms donor. NMR and induced circular dichroism (CD) experiments provide strong evidence of glucose and galactose encapsulation by **115**. Interestingly, the chirality of the sugar can be transferred to **115** upon binding, hence strong induced CD symmetrical and inverted signals are observed with the l‐ or d‐monosaccharide. This is a result of the preferential formation of one helical complex. Nevertheless, no induced CD signals or NMR shift changes are observed for mannose, concluding that no binding is established with this one. Replacing one appendage group with a DMAP moiety, Jeong and coworkers prepared a nucleophilic catalyst **116** for the regioselective acetylation of monosaccharides.^[^ ^128^ ^]^ More in details, foldamer bears at one end the catalytic DMAP unit; a triisopropylsilyl (TIPS) group is attached at the other end to modulate the solubility in organic solvents. **116** is a suitable catalyst for the selective acylation of octyl β‐d‐glucopyranoside (Figure 25b). **116** displays an association constant for this monosaccharide of 1.9 × 10^5^ m ^−1^ in dichloromethane. The acetylation reaction was performed in presence of DMAP or **116** as catalysts. From the comparison emerged that DMAP does not seem to be a selective catalyst, providing monoacetylated product distribution 6‐OAc: 4‐OAc: 3‐OAc: 2‐OAc = 34: 24: 41: 2. On the other hand, when **116** was used in the same reaction conditions the distribution of monoacetylated products is 6‐OAc: 4‐OAc: 3‐OAc: 2‐OAc = 90: 3: 6: 1. Recently the same group employed a similar type of indolocabazole‐based foldamer for the preparation of synthetic receptors for monosaccharides exploiting the principles of complexation‐driven equilibrium shifting and adaptive folding.^[^ ^129^ ^]^ This approach consists of the quantitatively assembly of monomeric receptor units in the presence of guests, by the formation of dynamic covalent bonds under reversible conditions (Figure 26). Jeong et al. discovered that diimine **119** can be prepared from foldamer **117**, benzene‐1,3‐diamine **118** and chloroacetic acid as an acid catalyst, in DMSO‐d_6_/CD_2_Cl_2_.^[^ ^129^ ^]^ The equilibrium is completely shifted toward **119** thanks to the presence of monosaccharides as guests, resulting in 97% isolated yield of **119**, compared to a 43% yield achieved without any guest. Furthermore, the authors found that the system is extremely selective for d‐galactose among other monosaccharides. Indeed, competition studies were done preparing mixed solutions of galactose, glucose, fructose or mannose, but only the solutions containing d‐galactose among other monosaccharides provide a well‐resolved spectrum matching the spectrum of **113** and d‐galactose as a single guest. Another interesting aspect is that d‐galactose is completely converted to its α‐d‐galactofuranose isomer (α‐d‐gf), after the binding, providing the complex **119**‐α‐d‐gf (Figure 26). Therefore, **119** self‐assembles thanks to the presence of the sugar and the sugar interconverts entirely into the single isomer which is capable to fit in the cavity; this constitutes a dual equilibrium shift. **119** shows an association constant toward α‐d‐galactofuranose of 5.40 × 10^4^ m ^−1^ and the crystals confirmed the presence of a tubular cavity where d‐galactose and a water molecule are incapsulated. The diimine **119** folds into a left‐handed helix with three turns. Replacing benzene‐1,3‐diamine **118** with 9H‐fluorene‐2,7‐diamine **120**, **121** can be obtained in the presence of different monosaccharides.^[^ ^129^ ^]^ In this case, the authors only observed the adaptive folding in the presence of methyl β‐d‐glucopyranoside (me‐β‐d‐glc) and methyl β‐d‐galactopyranoside (me‐β‐d‐gal), with some exceptional behaviors. The complexes formed exist in a dimeric form with two identical cavities, which can accommodate one monosaccharide molecule.

**Figure 27:**
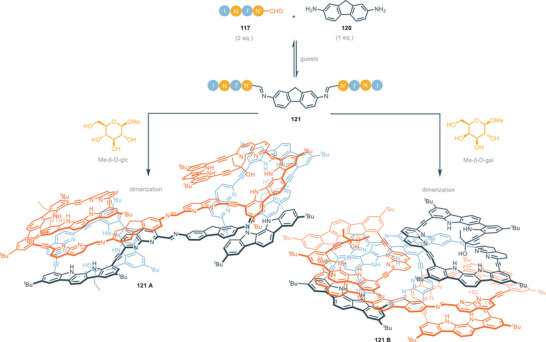
Molecular structures of the assembly of the dimeric foldamers **121A** and **121B**. In the case of **121A** each diimine strand of **121** folds with opposite orientation in the same plane of **119** hence, the dimerization takes place via face‐to‐face staking; on the other hand, in the case of **121B** the strands fold with the same orientation out of the plane hence, the dimerization takes place with a transoid geometry around the plane of **120**. Adapted from ref. [129].

Hence, 2:2 complexes **121A** and **121B** were identified. Further, the helical folding of **121A** (in the presence of (me‐β‐d‐glc)) is completely different from **121B** (in the presence of (me‐β‐d‐gal)). In the complexation of (me‐β‐d‐glc) the two helical strands assume opposite orientations; while in the case of (me‐β‐d‐gal) the two strands fold with the same orientation (Figure 27). The equilibrium related to the formation of the two complexes is controlled by temperature. Indeed, nearly equal signals of complex **121A** and **121B** are present at room temperature when prepared a solution of **121** with 1:1 molar ratio of (me‐β‐d‐glc) and (me‐β‐d‐gal). **121B** becomes predominant as the temperature decreases; on the other hand, raising the temperature favors the formation of **121A**, highlighting the unique behavior in selectivity and versatility of these synthetic foldamer‐based receptors. The binding data for compounds **115**, **116**, **119**, and **121A**–**121B** are summarized in Table 11.

**Table 11:** Association constants (m ^−1^) for compounds **115**, **116**, **119**, and **121A–121B** toward different guests. Unless specified, the titrations are performed via CD.

Receptor	*K*_A_ (d‐glucose)	K_A_ (d‐galactose)	*K*_A_ (d‐mannose)	*K*_A_ (octyl‐*β*‐d‐glucopyranoside)	*K*_A_ (*α*‐d‐gf)	*K*_A_ (Me‐β‐Glc)	*K*_A_ (Me‐ β‐Gal)	Ref.
**115** ^[^ ^a^ ^]^	9.6 × 10^4^	1.0 × 10^4^	≈0	‐	‐	‐	‐	^[^ ^127^ ^]^
**116** ^[^ ^b^ ^]^	‐	‐	‐	5.09 × 10	‐	‐	‐	^[^ ^128^ ^]^
**119** ^[^ ^c^ ^]^	‐	‐	‐	‐	5.40 × 10^4^	‐	^−^	^[^ ^129^ ^]^
**121A** ^[^ ^d^ ^]^	‐	‐	‐	‐	‐	3.16 × 10^13^	^−^	^[^ ^129^ ^]^
**121B** ^[^ ^d^ ^]^	‐	‐	‐	‐	‐		1.00 × 10^13^	^[^ ^129^ ^]^

^[a]^ In DMSO + 10% CH_2_Cl_2_.

^[b]^ In CH_2_Cl_2_.

^[c]^ In DMSO/CH_2_Cl_2_ (containing 0.04–0.06% H_2_O).

^[d]^ Determined via UV–vis titration in 5% (v/v) DMSO/CH_2_Cl_2_ (containing 0.04–0.06% H_2_O).

**Figure 28:**
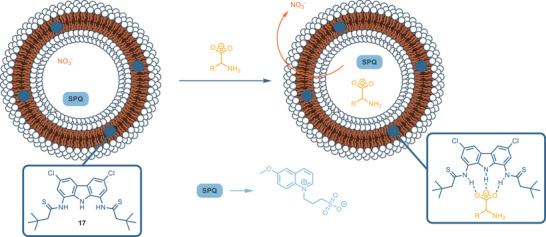
Dithioamide receptor **17** used as AAs transporter across lipidic bilayers in the SPQ assay. Adapted from ref. [130].

Carbazole‐based systems have also been employed in the binding and transport of AAs. In 2022, Chmielewski and coworkers studied the transport ability of dithioamide receptor **17** toward proteinogenic AAs across the lipid bilayer.^[^ ^130^ ^]^ This compound was already studied as an anion transporter (see Section 2).^[^ ^60^ ^]^ While complex heteroditopic receptors are generally required to facilitate the transport of zwitterionic species across lipid bilayers, the monotopic **17** proved to be an excellent transporter for AAs at physiological pH in a Cu^2+^‐calcein assay.^[^ ^131^ ^]^ As expected, the activity of transporter **17** increases with the lipophilicity of the AA, being the highest for Phe. Nevertheless, even highly polar AAs, such as Ser, are effectively transported inside the vesicles. The interaction only occurs with the anionic AA, while no effective transport was observed for the AA in their zwitterionic form, despite the zwitterionic species accounting for 97% of the AAs population at physiological pH. To investigate the transport properties of proteinogenic AAs at higher pH, the group developed a new assay based on the use of 6‐methoxy‐*N*‐(3‐sulfopropyl)quinolinium (SPQ), whose fluorescence is quenched by several deprotonated AAs and is unaffected by their zwitterionic counterparts (Figure 28).^[^ ^130^ ^]^ This assay revealed the same transport trend observed for the Cu^2+^‐calcein assay, although with a much higher diffusion rate, as expected. The presence of the AA inside the vesicle was further confirmed by ^13^C NMR experiment. This work paves the way for new strategies in the development of novel monotopic AA carriers which could be active at high pH, where traditional heteroditopic receptors are generally less effective.

**Figure 29:**
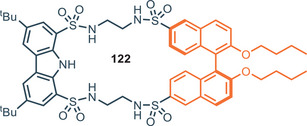
BINOL‐based chiral macrocycle for the enantioselective extractions of aromatic AAs.

Carbazole‐based chiral receptors have been used for the enantioselective extraction of aromatic AAs. For this purpose, Alcazar and coworkers developed macrocycle **122**, featuring a 1,8‐disulfonamide carbazole platform linked with a chiral binaphthyl unit. (Figure 29).^[^ ^132^ ^]^ The receptor was tested in the liquid–liquid extraction of zwitterionic AAs in CDCl_3_ from an aqueous phase. The system was able to extract quantitatively aromatic AAs (Phe and PheGly) in a highly enantioselective fashion (for PheGly, ee = 92%) and with high selectivity, since other AAs were not extracted at all.

## 4 Summary and Outlook

In summary, this review highlights the most relevant advances in carbazole‐based receptors as versatile, modular and efficient hosts for the recognition of different biologically relevant species, both charged (i.e., anions) and neutral (e.g., carbohydrates, xanthines). The structural tunability of the carbazole allows the introduction of several moieties, such as (thio)ureas, imines, sulfonamides and anthracenes, which could further assist the binding process via synergistic noncovalent interactions (e.g., hydrogen bonding, hydrophobic interactions, CH–π). Further expansion of the rigid and planar skeleton leads to indolocarbazoles, which have been used as building blocks for the preparation of synthetic foldamers capable of binding anions and carbohydrates.

Observing the binding properties toward anions, carbazole‐based receptors typically show greater affinity for polyatomic oxoanions, although exceptions exist (i.e., 1,8‐disulfonamide‐carbazole derivatives). Modifying the carbazole scaffold with different frameworks may help create receptors with varied cavity sizes, potentially enhancing their ability to bind smaller anions, such as chloride, which typically show lower binding affinities. Moreover, it would be interesting to more widely explore the introduction of non‐covalent interactions other than hydrogen‐bonding moieties, to improve the selectivity toward specific guest, in particular in the case of organic anions.^[^ ^78^ ^]^ While the planarity of carbazole provides an appealing framework for constructing synthetic receptors, it also presents challenges, mostly the generally low solubility of these compounds. This aspect limits their applications in different fields as well as the study of their properties. Although some strategies have been employed to mitigate this issue (e.g., introduction of alkyl chains or *t*‐butyl groups), more effort is desirable to face this complication. Additionally, the synthesis of these platforms remains challenging. Obtaining 1,8‐diaminocarbazole requires harsh nitration conditions, and functionalization at positions 3 and 6 remains limited, despite its role in tuning key properties. Additionally, the synthesis of 1,10‐substituted indolocarbazoles is hindered by the failure of the double Fischer indole route with strong electron‐withdrawing groups, making 1,10‐diamino‐indolocarbazoles inaccessible due to the difficulty of preparing the 1,10‐dinitro precursor. Furthermore, while carbazole‐based receptors have been extensively studied as sensors and transmembrane transporters, their application as supramolecular catalysts remain largely unexplored.

## Conflict of Interests

The authors declare no conflict of interest.
